# Cuproptosis and ferroptosis: signal pathways, diseases and therapeutic targets

**DOI:** 10.1038/s41392-026-03043-2

**Published:** 2026-09-23

**Authors:** Yuan-Yuan Li, Franklin R. Tay

**Affiliations:** 1 State Key Laboratory of Oral and Maxillofacial Reconstruction and Regeneration, National Clinical Research Center for Oral Diseases, Shaanxi Key Laboratory of Stomatology, Department of Prosthodontics, School of Stomatology, The Fourth Military Medical University, Xi’an, Shaanxi China; 2 Department of General Dentistry, Xiamen University Affiliated Chenggong Hospital, The 73rd Army Hospital of Chinese PLA, Xiamen, Fujian China; 3 Dental College of Georgia, Augusta University, Augusta, GA USA

**Keywords:** Target validation, Drug development, Drug development

## Abstract

Regulated cell death is essential for tissue homeostasis, and its dysregulation contributes to numerous human diseases. Cuproptosis and ferroptosis are metal-dependent forms of regulated cell death distinguished by different biochemical triggers and pathological consequences. Cuproptosis arises from copper-mediated disruption and aggregation of lipoylated mitochondrial proteins, whereas ferroptosis is driven by iron-dependent phospholipid peroxidation. Despite these mechanistic differences, the two pathways intersect through mitochondrial metabolism, redox imbalance, iron–sulfur cluster biology, and organelle crosstalk. Lysosomes, mitochondria, and the endoplasmic reticulum act as critical regulatory hubs that influence cellular susceptibility to both death modalities. This review summarizes current understanding of the molecular mechanisms governing cuproptosis and ferroptosis. It examines the genetic, epigenetic, transcriptional, post-transcriptional, and protein-level networks that regulate these processes. Evidence linking cuproptosis and ferroptosis to cardiovascular, neurodegenerative, autoimmune, metabolic, oral, infectious, and neoplastic diseases is critically evaluated. Therapeutic approaches are discussed, including metal ionophores, chelators, small-molecule modulators, nanomedicine-based delivery systems, and rational combination strategies. Particular attention is given to the context-dependent consequences of activating or suppressing these pathways, and to the challenge of selectively targeting diseased tissues without disrupting systemic metal homeostasis. Successful clinical translation will require reliable biomarkers, mechanistically informed patient stratification, tissue-selective delivery, and a clearer understanding of interactions between metal metabolism, immune responses, and treatment resistance. The review further identifies unresolved questions concerning pathway specificity, temporal regulation, biomarker validation, and the clinical safety of systemic or prolonged therapeutic modulation. Integrating these concepts may enable more precise exploitation of cuproptosis and ferroptosis as therapeutic targets across diverse diseases.

## Introduction

For decades, apoptosis was regarded as the principal form of regulated cell death (RCD), representing a genetically programmed process essential for normal development and the elimination of damaged cells.^1,2^ Over the past two decades, however, research has revealed a substantially more diverse spectrum of cell death modalities, each characterized by distinct molecular machinery, initiating stimuli, and physiological consequences. The identification of alternative RCD pathways, including necroptosis, pyroptosis, and autophagy-dependent cell death, has expanded current understanding of cell fate regulation and its contribution to disease pathogenesis.^3,4^ Many of these pathways are classified as forms of regulated necrosis and challenge the traditional distinction between programmed apoptotic death and accidental necrotic death.^5^ Collectively, these discoveries demonstrate that necrosis can be tightly regulated and potentially targeted pharmacologically.

Transition metals such as copper and iron are indispensable for numerous biological processes, including mitochondrial respiration and enzymatic catalysis. Their intracellular concentrations must be tightly regulated because both deficiency and excess impair cellular function.^6,7^ Disruption of metal homeostasis can provoke oxidative stress, mitochondrial dysfunction, and ultimately cell death, leading to the recognition of distinct forms of metal-dependent RCD.^8^ Cuproptosis, a recently identified form of copper-dependent cell death, was first described in 2022.^9^ Excess intracellular copper binds directly to lipoylated protein components (i.e., proteins modified by attachment of lipoic acid) within the mitochondrial tricarboxylic acid (TCA) cycle.^9^ Lipoic acid is a sulfur-containing cofactor that becomes covalently attached to certain proteins, especially mitochondrial enzyme complexes. This interaction promotes aggregation of essential metabolic enzymes, induces proteotoxic stress, and causes the loss of iron–sulfur (Fe–S) cluster-containing proteins, ultimately resulting in mitochondrial dysfunction and cell death.^9^

Ferroptosis, first described in 2012, was the earliest metal-dependent cell death pathway to be characterized in detail. It is an iron-dependent, non-apoptotic form of cell death caused by the excessive accumulation of lipid peroxides.^10^ Peroxidation is an oxidation reaction in which molecules are converted into peroxides or hydroperoxides. In ferroptosis, lipid peroxidation produces unstable lipid peroxides that compromise membrane structure and function, ultimately resulting in cell death. A central feature of this process is the failure of cellular antioxidant defenses, particularly the glutathione (GSH)-dependent enzyme glutathione peroxidase 4 (GPX4), which normally detoxifies phospholipid hydroperoxides.^11^ Ferroptosis has attracted substantial attention because of its involvement in ischemia–reperfusion injury, neurodegeneration, and cancer.^12,13^

Both copper and iron are well-established catalysts of Fenton-type chemistry. They can promote the decomposition of hydrogen peroxide to generate highly reactive free radicals, particularly hydroxyl radicals, that damage cellular biomolecules.^14,15^ These shared redox properties raise an important conceptual question: if both metals can catalyze radical-mediated damage, why is ferroptosis defined by iron-dependent lipid peroxidation, whereas cuproptosis is governed primarily by copper-dependent aggregation of lipoylated mitochondrial proteins instead of copper-induced lipid peroxidation?

The identification of cuproptosis and ferroptosis has expanded the growing family of RCD pathways and emphasized the distinct forms of toxicity associated with different transition metals. Dysregulated copper and iron metabolism is linked to a broad spectrum of human diseases, ranging from neurodegenerative disorders to common malignancies. This makes metal-dependent cell death an important area of investigation. A central question is how these apparently distinct pathways interact at the molecular level to determine cell fate.

This review provides an integrated perspective on cuproptosis and ferroptosis as representative forms of metal-dependent RCD. It first establishes a conceptual framework for metal-mediated cell death and then examines the molecular mechanisms and signaling cascades that connect disrupted metal homeostasis with their distinct execution pathways. Particular attention is devoted to the coordination of death signals through organelle crosstalk. The review also addresses the multilayered regulatory networks operating at the genetic, epigenetic, transcriptional, post-transcriptional, and protein levels that determine cellular susceptibility to cuproptosis and ferroptosis. Their pathophysiological roles are examined in cardiovascular, neurodegenerative, autoimmune, metabolic, oral, infectious, and neoplastic diseases. Therapeutic strategies targeting these pathways, including small-molecule modulators, nanomedicine, and combination approaches, are evaluated together with the principal challenges and future directions relevant to clinical translation. By integrating recent advances, this review clarifies the complex biology of metal-dependent cell death and its potential value as a source of therapeutic targets.

## Conceptual framework: metal-dependent RCD

Metal-dependent RCDs comprise a group of metabolically linked death programs in which disruption of the homeostasis or biological function of a specific metal cofactor impairs essential metabolic pathways and ultimately causes cellular collapse.^16^

Cuproptosis is defined by its dependence on copper. Unlike ferroptosis, it is not primarily executed through reactive oxygen species (ROS) accumulation or lipid peroxidation. Instead, excess copper directly binds to lipoylated proteins within the mitochondrial TCA cycle. This interaction promotes protein aggregation, induces proteotoxic stress, and initiates a downstream cascade characterized by the loss of Fe–S cluster-containing proteins.^17^

Ferroptosis is defined by its dependence on iron. It is executed through iron-catalyzed Fenton chemistry, which promotes the peroxidation of polyunsaturated fatty acids within cellular membranes.^13^ This mechanism distinguishes ferroptosis from other forms of RCDs because it does not rely on caspases, as in apoptosis, or on receptor-interacting protein kinase 3 (RIPK3) and mixed lineage kinase domain-like protein (MLKL), as in necroptosis. Instead, ferroptosis can be specifically inhibited by iron chelators and lipophilic antioxidants.^18^

Other metals have also been implicated in RCD, although the corresponding pathways are less clearly defined. Zinc overload can induce a form of regulated necrosis termed lysozincrosis.^5^ The recently described “disulfidptosis” occurs during glucose starvation in cells with high expression of solute carrier family 7 member 11 (SLC7A11).^19^ Under these conditions, cystine accumulation induces disulfide stress, causing collapse of the actin cytoskeleton and cell death.^19^ Although this process is associated with metabolic dysregulation, it is not directly mediated by a metal catalyst in the same manner as cuproptosis or ferroptosis. A distinct cobalt-dependent pathway, “cobaltosis,” has also been proposed, although rigorous experimental validation is still required.^20^ These additional metal-associated or metabolically regulated cell death pathways are beyond the scope of the present review.

## Molecular mechanisms and signaling pathways

### Dysregulation of metal homeostasis: a common trigger for cell death

**Fig. 1:**
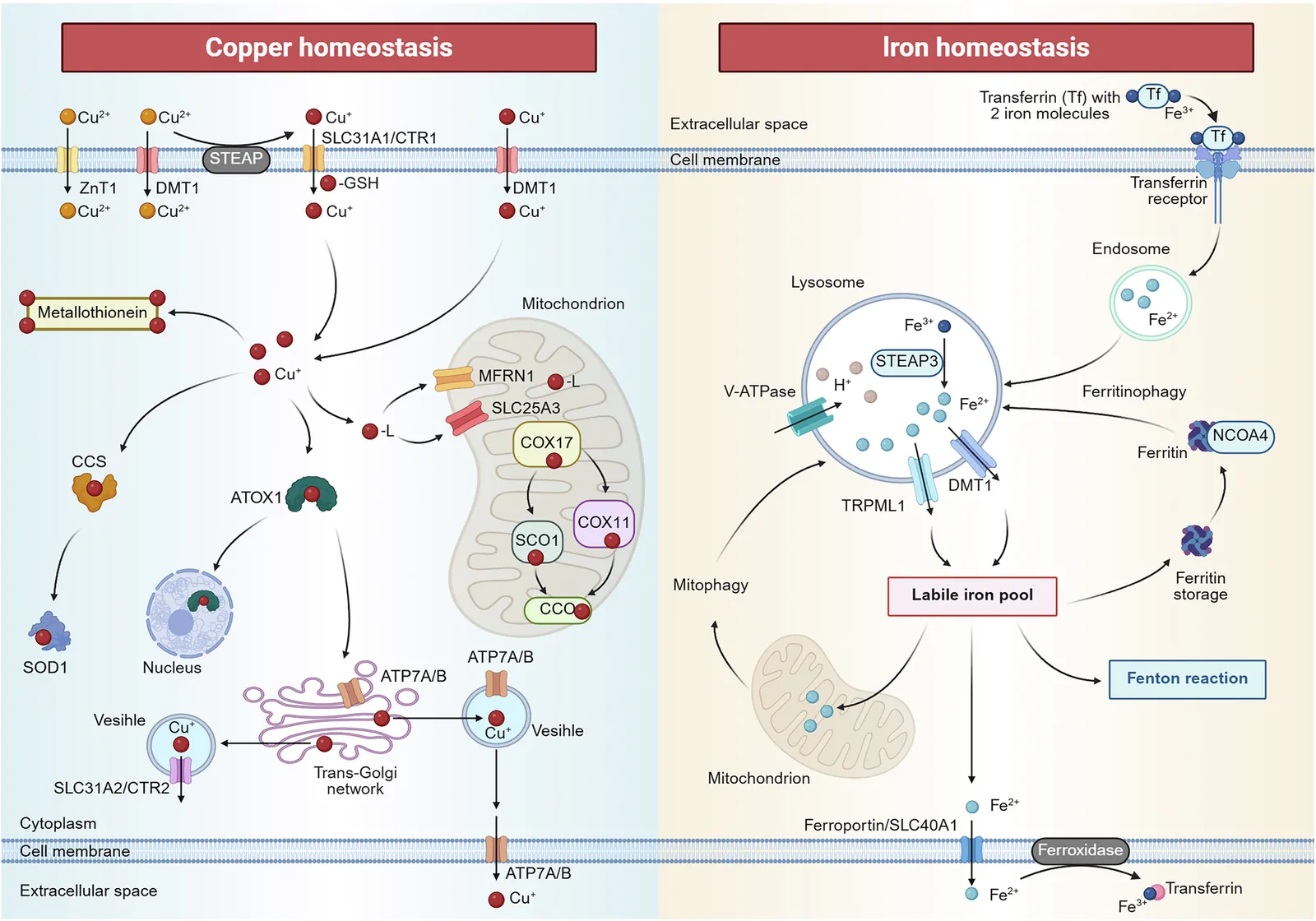
Copper and iron homeostasis. The left panel summarizes the major routes of intracellular copper handling. The right panel summarizes intracellular iron metabolism. Based on data from published papers.^34,45^ MFRN1 mitoferrin-1. Created in BioRender. Li, Y. (2026) https://BioRender.com/pc22unu

Both cuproptosis and ferroptosis arise from disruption of the tightly controlled intracellular balance of copper and iron. Cells therefore maintain coordinated systems for the uptake, trafficking, storage, and detoxification of these essential yet potentially toxic metals (Fig. 1).^21,22^

Copper is an essential cofactor for enzymes involved in several fundamental biological processes. These processes include mitochondrial respiration through cytochrome c oxidase, antioxidant defense through superoxide dismutase 1 (SOD1), and iron metabolism through ceruloplasmin.^23–25^ Cellular copper homeostasis is maintained by a coordinated network of transporters and chaperones that regulate copper uptake, intracellular distribution, sequestration, and export. In the extracellular environment, cupric ions (Cu^2+^) are reduced to cuprous ions (Cu^+^) by metalloreductases of the six-transmembrane epithelial antigen of prostate (STEAP) family (Fig. 1).^26^ Cu^+^ enters the cell primarily through the high-affinity transporter copper transporter 1 (CTR1, also known as solute carrier family 31 member 1 [SLC31A1]) (Fig. 1).^27^ Glutathione binds Cu^+^ within the CTR1 pore and facilitates its release into the cytosol.^28^ Zinc transporter 1 and divalent metal transporter 1 (DMT1) also mediate copper transport into cells (Fig. 1).^29,30^ Upon entering the cytosol, Cu is bound by specific chaperones that prevent toxicity and direct its intracellular distribution. Copper is distributed to several intracellular destinations, including the mitochondria, trans-Golgi network, and nucleus (Fig. 1).^31^ Copper chaperone for superoxide dismutase (CCS) delivers Cu to SOD1, whereas antioxidant 1 copper chaperone (ATOX1) transfers Cu to the adenosine triphosphatase copper transporters 7 A (ATP7A) and 7B (ATP7B) (Fig. 1).^32^ Within the mitochondria, copper is incorporated into cytochrome c oxidase (CCO), where it contributes to electron transport and redox activity.^33^ In the mitochondrial intermembrane space, cytochrome c oxidase copper chaperone 17 (COX17) binds copper and transfers it to synthesis of cytochrome c oxidase 1 (SCO1) or cytochrome c oxidase copper chaperone 11 (COX11), which subsequently deliver copper to specific CCO subunits.^34^ This process also depends on solute carrier family 25 member 3 (SLC25A3), a transporter located in the inner mitochondrial membrane.^35^ Mitoferrin-1, a mitochondrial iron transporter identified in fish, has also been reported to transport copper (Fig. 1).^36^ Copper efflux and delivery to secretory compartments are managed by ATP7A and ATP7B. These P-type adenosine triphosphatases (ATPases) transport Cu^+^ across membranes using the energy generated by adenosine triphosphate (ATP) hydrolysis and undergo Cu-dependent trafficking between the trans-Golgi network and the plasma membrane (Fig. 1).^32^ Within the trans-Golgi network, ATP7A and ATP7B transport cytosolic Cu^+^ into the lumen for incorporation into secreted cuproenzymes.^37,38^ In hepatocytes, ATP7B specifically delivers Cu^+^ to ceruloplasmin within the trans-Golgi network.^38^ When intracellular copper concentrations increase, excess copper is sequestered by metallothionein or exported through ATP7A- and ATP7B-mediated vesicular trafficking (Fig. 1).^39^ Copper transporter 2 (CTR2), also known as solute carrier family 31 member 2 (SLC31A2), facilitates copper mobilization from intracellular vesicular compartments (Fig. 1). Copper transporter 2 also promotes the generation of a truncated form of CTR1 that facilitates the release of copper from endocytic compartments into the cytosol.^40^ Disturbance of these pathways, whether caused by genetic defects such as ATP7B mutations in Wilson’s disease, or by environmental copper overload, increases labile intracellular copper pool and creates conditions favorable for cuproptosis.^41,42^

Iron is indispensable for oxygen transport by hemoglobin, deoxyribonucleic acid (DNA) synthesis through ribonucleotide reductase, and cellular energy metabolism through Fe–S cluster proteins in the electron transport chain.^43,44^ Cellular iron uptake occurs essentially through transferrin receptor-mediated endocytosis (Fig. 1). Ferric iron (Fe^3+^) is released from transferrin within the acidic lysosomal lumen and reduced to ferrous iron (Fe^2+^) by the STEAP3 reductase (Fig. 1).^45^ This bioavailable Fe^2+^ is then exported to the cytoplasm by transporters such as DMT1 and transient receptor potential channel mucolipin 1 (TRPML1), where it enters the labile iron pool and is chelated by GSH to limit its reactivity (Fig. 1).^45^ Cytoplasmic iron is subsequently used in metabolic processes, stored within ferritin nanocages, or exported by ferroportin (a.k.a. solute carrier family 40 member 1 [SLC40A1]) (Fig. 1).^46,47^ The export of iron from cells depends on the activity of ferroxidases including ceruloplasmin and hephaestin. These ferroxidases oxidize Fe^2+^ to Fe^3+^, thereby enabling its loading onto the circulating iron carrier transferrin (Fig. 1).^48^

Disruption of lysosomal acidification, such as through impaired proton-pumping vacuolar-type adenosine triphosphatase (V-ATPase), causes cellular iron starvation, mitochondrial dysfunction, and non-apoptotic cell death.^49,50^ Conversely, lysosomal iron overload induced by inhibition of DMT1 or by agents such as ironomycin triggers lipid peroxidation and lysosomal membrane permeabilization, culminating in ferroptosis.^51,52^ Autophagic pathways further regulate this process. Nuclear receptor coactivator 4 (NCOA4)-mediated ferritinophagy increases cytosolic labile iron and sensitizes cells to ferroptosis, whereas mitophagy of iron-rich mitochondria may also contribute to iron mobilization under stress conditions (Fig. 1).^53,54^ Excess labile iron, particularly Fe^2+^, then participates in Fenton chemistry and generates highly reactive hydroxyl radicals that initiate lipid peroxidation (Fig. 1).^45^

### Divergent execution mechanisms of cuproptosis and ferroptosis

Cuproptosis is closely associated with mitochondrial respiration and the metabolism of lipoylated enzymes, resulting primarily in proteotoxic stress instead of widespread membrane lipid damage.^34^ In contrast, ferroptosis is characterized by uncontrolled peroxidation of susceptible phospholipids that ultimately compromises membrane integrity.^55^

#### Cuproptosis

Intracellular copper concentrations are strictly controlled by a coordinated network of transporters and chaperones. Disruption of this balance causes copper overload and may trigger cuproptosis. An important upstream regulator is ferredoxin 1 (FDX1), which reduces Cu^2+^ to the more reactive Cu^+^ state. This enables copper to bind to the lipoylated components of mitochondrial enzymes.^42^ In this process, excess copper binds directly to lipoylated acyl-thioester proteins, most notably dihydrolipoamide S-acetyltransferase (DLAT), a component of the pyruvate dehydrogenase complex.^9^ This interaction promotes aggregation of DLAT and other lipoylated proteins, resulting in acute proteotoxic stress. A major downstream consequence is the destabilization and loss of Fe–S cluster-containing proteins, which impair mitochondrial respiration and other essential cellular functions, ultimately culminating in cell death.^42^ This mechanism distinguishes cuproptosis from other RCD pathways such as apoptosis or ferroptosis.^56,57^

Emerging evidence also indicates a distinct mitochondria-independent mechanism of cuproptosis. This alternative pathway is mediated primarily by the disulfiram metabolite bis(diethyldithiocarbamate)-copper complex (CuET). After formation in vivo, this complex preferentially accumulates in tumor tissues and exerts its cytotoxic effects without primarily targeting mitochondria.^58^ Mechanistically, CuET binds directly to nuclear protein localization protein 4 (NPL4), a cofactor of the p97 segregase complex. This interaction induces the rapid aggregation and immobilization of NPL4 within the nucleus and cytoplasm.^58,59^ Recruitment of p97 into these NPL4 aggregates disrupts the ubiquitin-proteasome system at a pre-proteasomal stage, resulting in the accumulation of poly-ubiquitinated proteins and activation of the unfolded protein and heat shock responses.^58,59^ Notably, the cytotoxicity of different dithiocarbamate/copper complexes correlates strongly with their ability to induce NPL4 aggregation, This feature establishes NPL4 as an important extra-mitochondrial effector of copper-dependent cell death.^59^

This mechanism differs from that of elesclomol, a small-molecule copper ionophore in which copper release within mitochondria is FDX1-dependent. However, elesclomol may also increase cytosolic copper availability, as indicated by the FDX1-independent trafficking of ATP7A and activation of SOD1 after elesclomol–copper treatment. These observations indicate that elesclomol can release copper through an additional non-mitochondrial route.^60^

#### Ferroptosis

The execution of ferroptosis is governed by a complex, multilayered regulatory network. Its central mechanism reflects disruption of the balance among iron metabolism, lipid metabolism, and antioxidant defenses.^61^

**Fig. 2:**
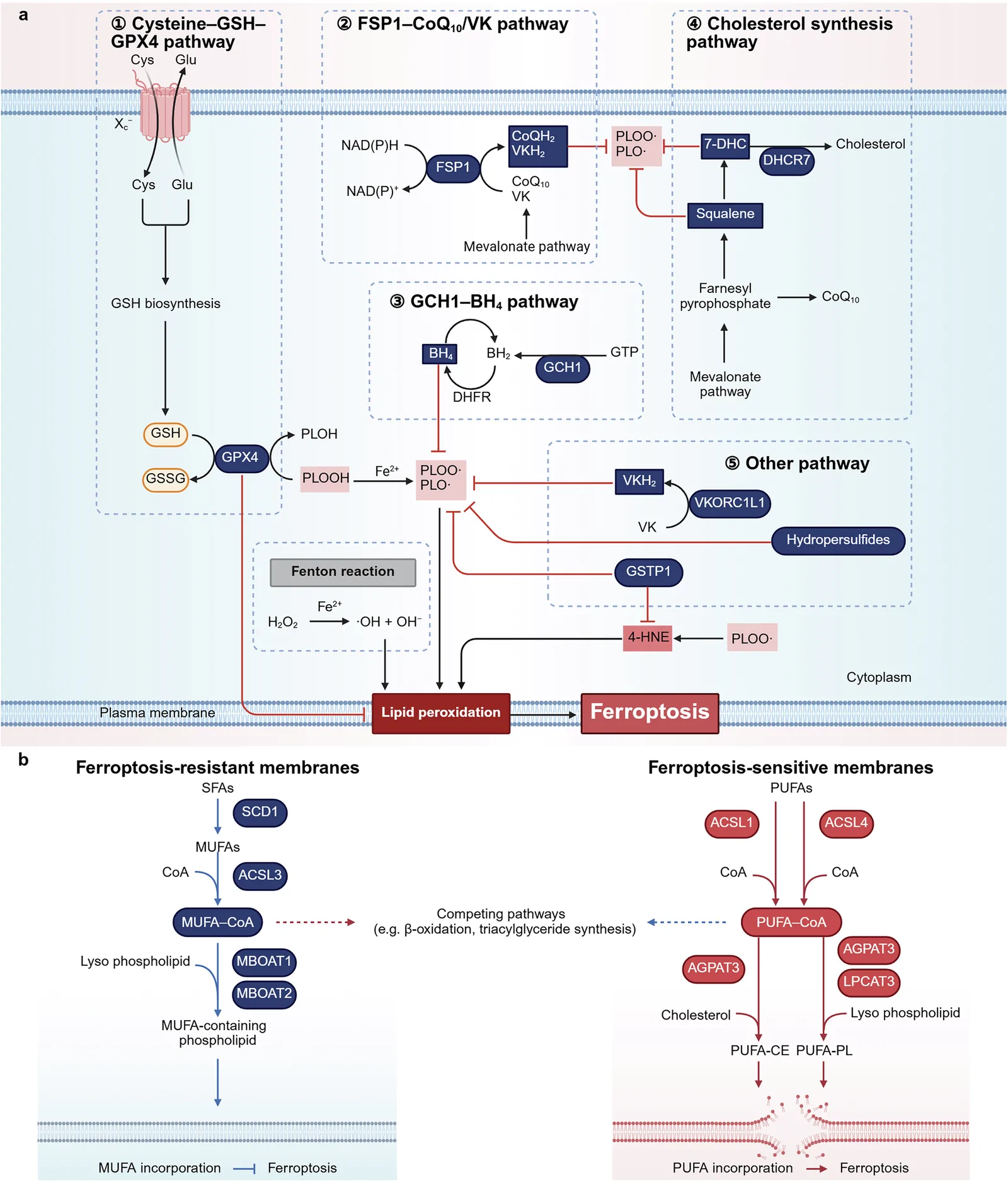
Mechanisms of ferroptosis. **a** Ferroptosis-suppressing pathways. **b** Effect of membrane phospholipid composition on cellular vulnerability to ferroptosis. When PUFAs become esterified into phospholipids, cells acquire ferroptosis sensitivity. Conversely, incorporation of MUFAs establishes resistance to this lethal process. Based on data from published papers.^65,69,72,458^ 4-HNE 4-hydroxy-2-nonenal, 7-DHC 7-dehydrocholesterol, BH_2_ 7,8-dihydrobiopterin, BH_4_ tetrahydrobiopterin, CoA coenzyme A, CoQ_10_ coenzyme Q_10_, CoQH₂ ubiquinol, Cys cystine, DHCR7 7-dehydrocholesterol reductase, DHFR dihydrofolate reductase, Glu glutamate, GSSG glutathione disulfide, GTP guanosine triphosphate, MUFAs monounsaturated fatty acids, OH hydroxyl radicals, PLO phospholipid alkoxyl radical, PLOO phospholipid peroxyl radical, PLOOH phospholipid hydroperoxides, PLOH phospholipid alcohols, PUFAs polyunsaturated fatty acids, PUFA-CE PUFA-containing cholesterol esters, PUFA-PL PUFA-containing phospholipid, SFAs saturated fatty acids, VK vitamin K, VKH_2_ vitamin K hydroquinone. Created in BioRender. Li, Y. (2026) https://BioRender.com/pc22unu

Excess labile Fe^2+^ catalyzes the formation of lipid-damaging radicals from pre-existing lipid hydroperoxides via the Fenton reaction. In the classical Fenton reaction, Fe^2+^ is oxidized to Fe^3+^ whereas hydrogen peroxide is reduced to the hydroxyl radical and hydroxide ion: Fe^2+^ + H_2_O_2_ → Fe^3+^ + OH + OH^−^ (Fig. 2a). Hydroxyl radicals are highly reactive and rapidly oxidize nearby biomolecules. Iron also catalyzes the conversion of phospholipid hydroperoxides (PLOOH) to phospholipid alkoxyl radicals (PLO), thereby propagating the lipid peroxidation chain reaction (Fig. 2a).^62^ Continuous radical regeneration amplifies this chain reaction and promotes extensive lipid peroxidation (Fig. 2a).^63^ The substrates for this peroxidation are polyunsaturated fatty acids, particularly arachidonic acid and adrenic acid, that are esterified into membrane phospholipids.^64^

Acyl-coenzyme A synthetase long-chain family member 4 (ACSL4) catalyzes the attachment of coenzyme A to polyunsaturated fatty acids, producing activated fatty acyl-coenzyme A intermediates for phospholipid synthesis (Fig. 2b). Lysophosphatidylcholine acyltransferase 3 (LPCAT3) subsequently incorporates these activated fatty acids into membrane phospholipids. The resulting polyunsaturated fatty acid-containing phospholipids are highly susceptible to oxidation, increasing membrane vulnerability and cellular sensitivity to ferroptosis (Fig. 2b).^65,66^ Another acyl-coenzymes A synthetase, ACSL1, promotes the cellular uptake and activation of conjugated polyunsaturated fatty acids such as α-eleostearic acid, which is particularly susceptible to lipid peroxidation.^67^

1-acylglycerol-3-phosphate O-acyltransferase 3 (AGPAT3) promotes ferroptosis by catalyzing the incorporation of polyunsaturated fatty acids into the *sn*-2 position of ether phospholipids in the endoplasmic reticulum.^68^ In cells deficient in solute carrier family 47 member 1 (SLC47A1), sterol O-acyltransferase 1 (SOAT1) promotes ferroptosis through a pathway that bypasses lysophospholipid metabolism.^69^ Lipid peroxidation may proceed through enzymatic or non-enzymatic pathways. Lipoxygenases are iron-containing enzymes that directly oxygenate polyunsaturated fatty acids to form lipid hydroperoxides, thereby amplifying ferroptotic signaling.^70^

Monounsaturated fatty acids are less susceptible to oxidative damage.^71^ They compete with polyunsaturated fatty acids for incorporation into membrane phospholipids, thereby reducing membrane vulnerability to oxidative injury (Fig. 2b). Accordingly, supplementation with exogenous monounsaturated fatty acids such as oleate protects cells against ferroptosis through a mechanism dependent on ACSL3, which preferentially activates monounsaturated fatty acids.^72^ Resistance to ferroptosis is also promoted by enzymes that mediate monounsaturated fatty acid synthesis and their subsequent esterification into phospholipids. These enzymes include stearoyl-coenzyme A desaturase 1 (SCD1), membrane bound O-acyltransferase domain containing 1 (MBOAT1), and membrane bound O-acyltransferase domain containing 2 (MBOAT2) (Fig. 2b).^73,74^ Fatty acid synthase and acetyl-coenzyme A carboxylase 1 further influence ferroptosis resistance by mediating palmitate synthesis and the production of malonyl-coenzyme A from acetyl-coenzyme A, respectively. This regulatory function is particularly evident during energy stress.^75^

The function of LPCAT3 can nevertheless be context dependent. In mutant Kirsten rat sarcoma viral oncogene homolog (KRAS) lung cancer, suppression of the usually pro-ferroptotic LPCAT3 paradoxically increases ferroptosis sensitivity.^76^ Lipidomic and biochemical analyses indicate that under conditions of equal substrate availability, LPCAT3 preferentially incorporates the saturated fatty acid palmitate over polyunsaturated fatty acids such as arachidonic acid.^76^

Ferroptosis occurs when the rate of lipid peroxidation exceeds the cellular capacity for detoxification. The principal defense system is the GPX4 pathway (Fig. 2a). Glutathione peroxidase 4 is a selenoprotein. Its active site contains selenocysteine, which is essential for reducing phospholipid hydroperoxides.^77^ Biosynthesis of selenocysteine and its incorporation into GPX4 require a specialized selenium delivery system. Low-density lipoprotein receptor-related protein 8 (LRP8) imports selenium bound to selenoprotein P. Deficiency of this receptor promotes ferroptosis by impairing GPX4 translation.^78^ Peroxiredoxin 6 also functions as a selenium carrier. It transfers selenium to selenophosphate synthetase 2 and enhances selenocysteine-tRNA synthesis, thereby maintaining GPX4 abundance and ferroptosis resistance.^77,79,80^

Glutathione peroxidase 4 activity also depends on the availability of GSH (Fig. 2a). Glutathione synthesis requires cystine, which cells acquire primarily from the external milieu through the system x_c_^−^ (Fig. 2a).^81^ System x_c_^−^ comprises the functional component xCT (a.k.a. SLC7A11) and a structural component 4F2hc (a.k.a. solute carrier family 3 member 2 [SLC3A2]).^81^ The lethal consequences of impaired cystine uptake were observed in neuronal cells before the term “ferroptosis” was introduced. Glutamate-induced cytotoxicity in N18-RE-105 neuroblastoma-retina hybrid cells by inhibiting cystine transport through system x_c_^−^. This resulted in GSH depletion and intracellular peroxide accumulation.^82^ Similarly, glutamate‑induced death of PC-12 cells involved cystine deprivation and was prevented by the antioxidant vitamin E.^83^ Inhibition or depletion of GPX4 (e.g. by RAS-selective lethality protein 3) or depletion of its substrate GSH (e.g., by erastin-mediated inhibition of system x_c_^−^) are canonical inducers of ferroptosis.^84,85^

Recent studies have identified alternative thiol-containing molecules that can sustain GPX4 activity. Cysteine and homocysteine can serve as direct reducing substrates for GPX4 when GSH synthesis is impaired, thereby inhibiting ferroptosis independently of the GSH biosynthetic pathway.^86^ Similarly, N-acetylcysteine and its enantiomer d-N-acetylcysteine, which cannot be converted to cysteine, also function as bona fide reducing substrates for GPX4. In vitro enzymatic assays have demonstrated their ability to reduce phosphatidylcholine hydroperoxide in a GPX4-dependent manner.^87^ The anti-ferroptotic effects of these alternative substrates strictly require GPX4 because they cannot prevent cell death following GPX4 deletion or complete inhibition.^86,87^ Common reducing agents such as β-mercaptoethanol can also act as GPX4 substrates in cell-free systems, although their cellular activity varies due to differences in permeability and stability.^87^ Evolutionary analyses further suggest that GSH became the dominant GPX4 substrate through adaptation to aerobic metabolism. This is because ancestral GPX enzymes from organisms lacking GSH synthesis exhibit greater binding affinity for cysteine and homocysteine.^86^

Cells have also evolved other parallel defense systems that suppress ferroptosis independently of GPX4 (Fig. 2a). One such system involves ferroptosis suppressor protein 1 (FSP1).^88^ Using nicotinamide adenine dinucleotide phosphate, reduced form (NADPH), FSP1 reduces extramitochondrial ubiquinone (coenzyme Q_10_) and supplemented vitamin K into radical-trapping antioxidants that suppress lipid peroxidation independently of GPX4 (Fig. 2a).^88^ Coenzyme Q_10_ is synthesized through the mevalonate pathway, which generates the intermediate metabolites isopentenyl pyrophosphate and dimethylallyl pyrophosphate.^89^ These intermediates act as precursors for the synthesis of vitamin K, cholesterol, and coenzyme Q_10_. Within this pathway, mevalonate diphosphate decarboxylase catalyzes the conversion of mevalonate diphosphate to isopentenyl pyrophosphate.^89^ Inhibition of the mevalonate pathway with atorvastatin or 6-fluoromevalonate significantly reduces intracellular coenzyme Q_10_ concentrations, compromises the FSP1/coenzyme Q_10_ antioxidant system, and increases ferroptosis sensitivity.^89^

Another protective pathway involves guanosine triphosphate cyclohydrolase-1 (GCH1) and its metabolite tetrahydrobiopterin. Tetrahydrobiopterin levels are maintained by dihydrofolate reductase, which reduces dihydrobiopterin back to tetrahydrobiopterin (Fig. 2a). Tetrahydrobiopterin functions as a radical-trapping antioxidant that suppresses lipid peroxidation and prevents ferroptosis independently of both GPX4 and GSH (Fig. 2a).^90,91^ This pathway is especially relevant in certain types of cancer, in which its inhibition increases cellular sensitivity to ferroptosis.^92^

A more recently identified protective mechanism involves the accumulation of 7-dehydrocholesterol, which functions as a sacrificial antioxidant by scavenging lipid radicals and suppressing phospholipid peroxidation (Fig. 2a).^93^ Glutathione S-transferase P1 (GSTP1) limits ferroptotic damage by detoxifying 4-hydroxy-2-nonenal, a toxic byproduct of lipid peroxidation, and by directly reducing lipid hydroperoxides to their corresponding non-toxic lipid alcohols (Fig. 2a).^94^ Vitamin K epoxide reductase complex subunit 1 like 1 (VKORC1L1) has also been identified as a potent ferroptosis repressor.^95^ This enzyme functions independently of the canonical GPX4 and FSP1 pathways by generating reduced vitamin K, which acts as a radical-trapping antioxidant to counter phospholipid peroxidation (Fig. 2a).^95^ Notably, VKORC1L1 is a direct transcriptional target of p53, which connects this ferroptosis defense pathway to tumor suppressor signaling. Its inhibitor warfarin has therefore attracted interest as a potential anti-cancer agent.^95^

Enzymatically produced sulfane sulfur species, particularly hydropersulfides, also function as potent radical-trapping antioxidants. These species suppress lipid peroxidation and ferroptosis independently of GPX4.^96,97^ Hydropersulfides scavenge phospholipid-derived peroxyl radicals at rates comparable to those of potent ferroptosis inhibitors. They also terminate radical chain reactions through perthiyl radical formation and self-recombination.^96^ Cysteine contributes to this protective pathway by donating sulfur for sulfane sulfur biosynthesis, thereby facilitating GPX4-independent ferroptosis resistance.^97^ Ferroptosis ensues when these protective mechanisms can no longer restrain lipid peroxidation (Fig. 2a).

### Organelle interfaces and molecular crosstalk in cuproptosis and ferroptosis

Cuproptosis and ferroptosis are not executed in isolation. Instead, their initiation and progression depend on coordinated interactions among multiple organelles that function as signaling hubs, metabolic centers, and sources of cell death-inducing molecules.

#### Mitochondrial regulation of cuproptosis and ferroptosis

**Fig. 3:**
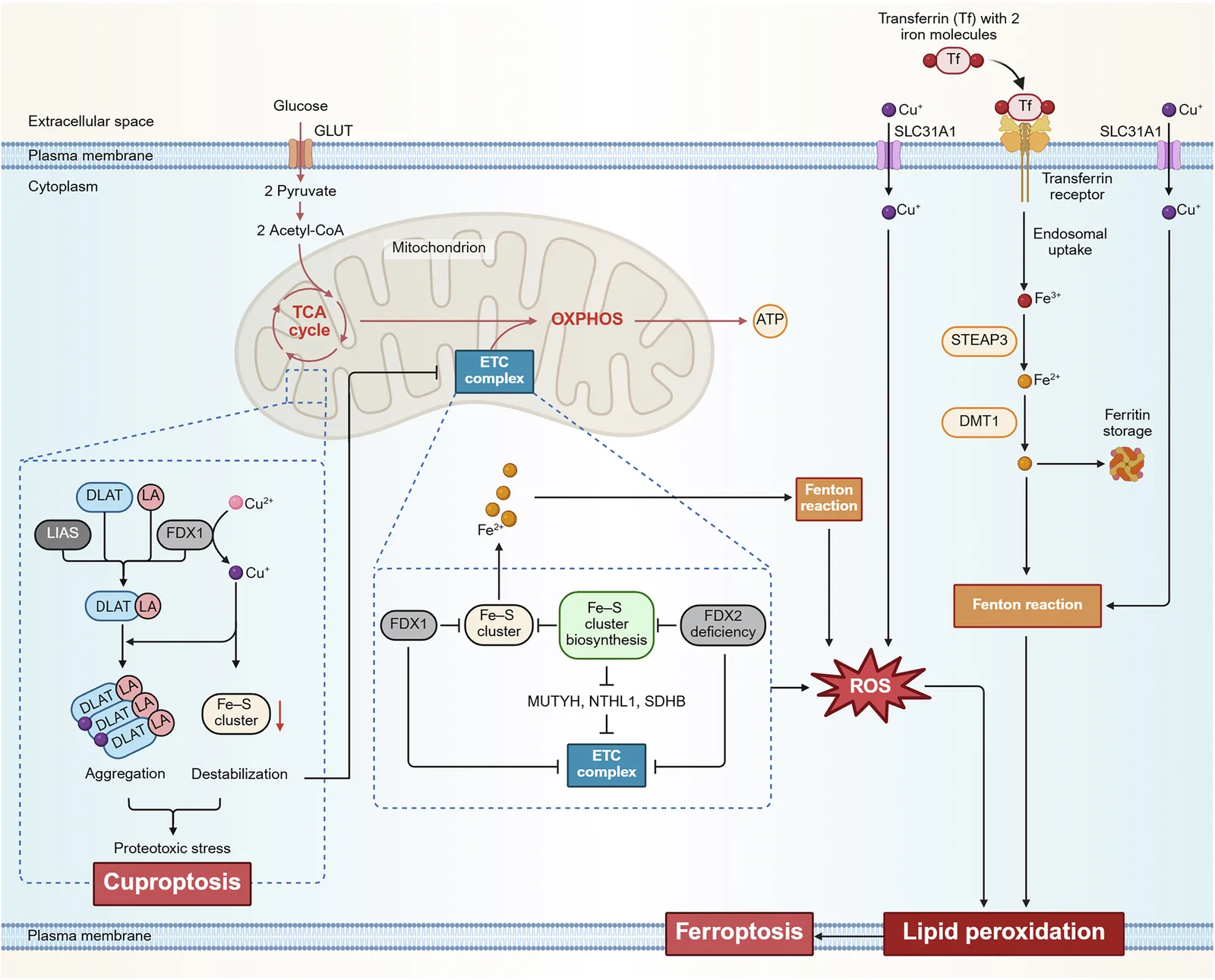
Mitochondrion as the organelle where cuproptosis and ferroptosis intersect. Acetyl-CoA acetyl coenzyme A, ETC electron transport chain, GLUT glucose transporter, LA lipoic acid, OXPHOS oxidative phosphorylation, Tf transferrin. Created in BioRender. Li, Y. (2026) https://BioRender.com/pc22unu

Mitochondria occupy a central position in both cuproptosis and ferroptosis, although their roles differ between the two pathways. The overall state of mitochondrial function strongly influences cellular susceptibility to both forms of cell death (Fig. 3).

Mitochondria are the principal site of cuproptosis. The process is characterized by the accumulation of copper in the mitochondrial matrix and the direct targeting of lipoylated TCA cycle enzymes (Fig. 3).^98,99^ Mitochondrial integrity and metabolic activity therefore represent both the target and the determinant of cuproptosis. Factors that increase dependence on mitochondrial metabolism, such as treatment of prostate cancer cells with the androgen receptor antagonist enzalutamide, can increase susceptibility to copper-induced cell death.^100^

The mitochondrial proteins FDX1 and ferrodoxin 2 (FDX2) have emerged as important regulators of metal-dependent cell death. Ferrodoxin 1 promotes cuproptosis through copper reduction, whereas FDX2 deficiency promotes ferroptosis by disrupting Fe–S cluster homeostasis. These features highlight the involvement of both proteins at the interface between mitochondrial metabolism and cell death.^101^ Together with ferredoxin reductase, FDX1 and FDX2 form a dedicated mitochondrial electron-transfer system required for several biochemical processes, including the TCA cycle, oxidative phosphorylation, and Fe–S cluster synthesis (Fig. 3).^101^

Within the mitochondria, FDX1 reduces Cu^2+^ to Cu^+^, enabling copper to bind to lipoylated motifs in DLAT. This interaction promotes DLAT oligomerization and destabilizes Fe–S cluster-containing proteins (Fig. 3).^42^ Ferrodoxin 2 contributes to ferroptosis regulation by serving as the principal electron donor for mitochondrial [4Fe–4S] cluster biogenesis (Fig. 3).^102^ Accordingly, FDX2 dysfunction impairs Fe–S cluster synthesis (Fig. 3).^103^ This defect reduces mitochondrial iron sequestration, causes mitochondrial iron overload, and decreases the abundance of Fe–S proteins.^104^ These proteins include succinate dehydrogenase complex iron–sulfur subunit B (SDHB), a component of the mitochondrial electron transport chain, and the DNA repair glycosylases mutY DNA glycosylase (MUTYH) and nth like DNA glycosylase 1 (NTHL1) (Fig. 3).^104^

The resulting accumulation of Fe^2+^ promotes lethal lipid peroxidation through Fenton chemistry, whereas the loss of Fe–S proteins, including electron transport chain components, further intensifies oxidative stress (Fig. 3).^104^ Ferredoxin 2 deficiency may also activate compensatory pathways that increase cellular iron uptake, including upregulation of the metal transporter SLC39A14.^105^ In contrast, direct involvement of FDX2 in cuproptosis has not yet been demonstrated.^101^

The role of mitochondria in ferroptosis is more context dependent but nevertheless important. Mitochondria are a major source of cellular ROS; electron leakage from the respiratory chain can contribute to lipid peroxidation. Mitochondrial dysfunction may therefore intensify oxidative stress and promote ferroptosis (Fig. 3).^106^ Mitochondrial metabolism, particularly glutaminolysis and the TCA cycle, also supplies metabolites and energy that influence ferroptosis sensitivity. For example, depletion of GSH increases cellular reliance on mitochondrial respiration, whereas electron transport chain inhibitors may either promote or suppress ferroptosis depending on the biological context.^55^ Mitochondrial dynamics also contribute to ferroptosis regulation. Excessive mitochondrial fission is frequently observed during ferroptosis and may amplify mitochondrial injury and ROS production.^106^

#### Lysosomal regulation of metal handling and lipid peroxidation

**Fig. 4:**
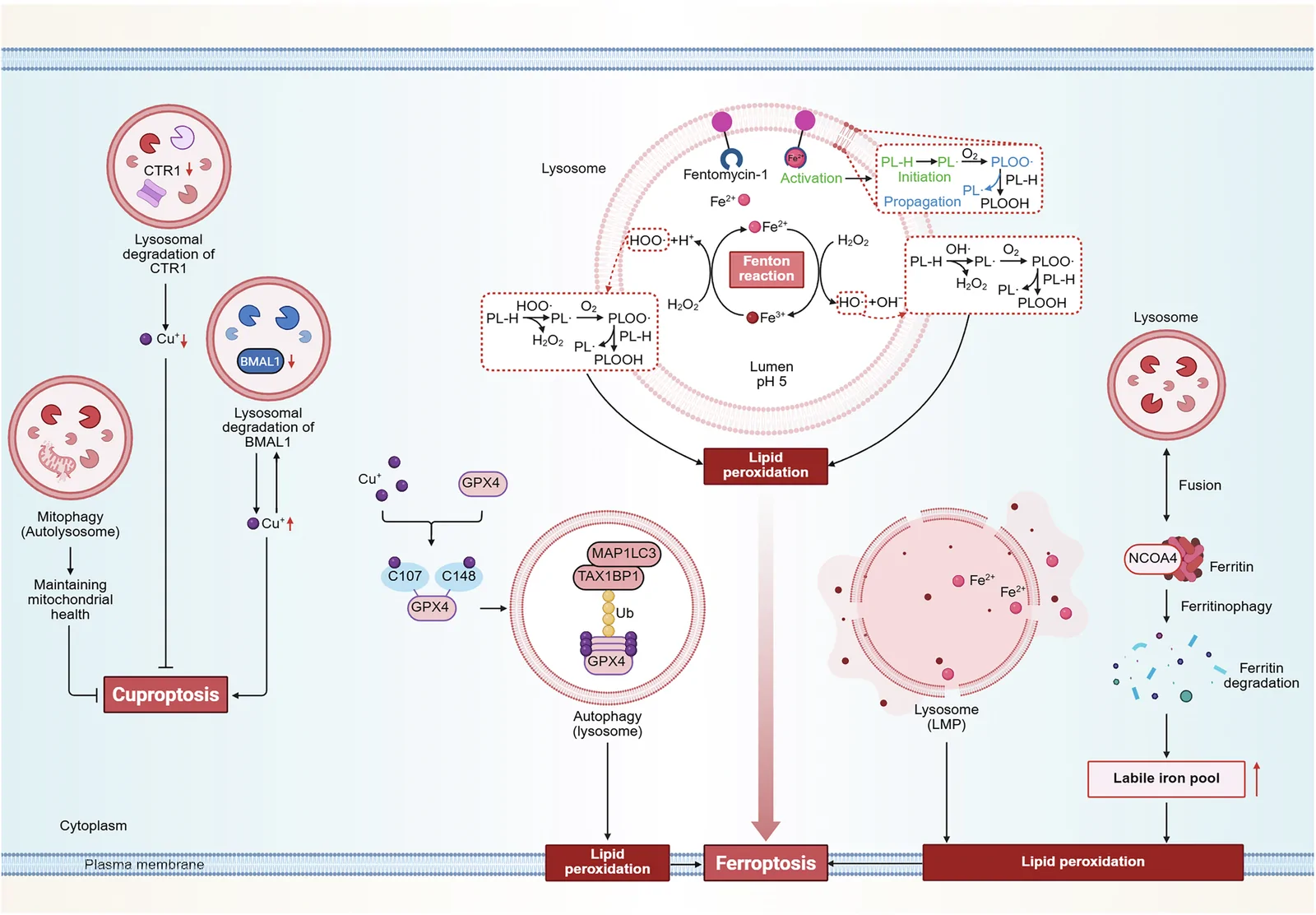
Roles of lysosomes in cuproptosis and ferroptosis. LMP lysosomal membrane permeabilization, HO hydroxyl radical, HOO hydroperoxyl radical, MAP1LC3 microtubule-associated protein 1 light chain 3, PL phospholipid radical, PL-H phospholipid, PLOO phospholipid peroxyl radical, PLOOH phospholipid hydroperoxides, Ub ubiquitination. Created in BioRender. Li, Y. (2026) https://BioRender.com/pc22unu

Recent evidence identifies lysosomes as important regulators of cuproptosis. Their degradative functions may either suppress or promote cuproptosis, depending on the cellular context and the proteins selected for degradation. Phillygenin promotes the lysosomal localization and degradation of CTR1, thereby reducing intracellular copper accumulation and protecting cardiomyocytes from cuproptosis (Fig. 4).^107^ In glioblastoma stem cells, in contrast, copper induces p62-mediated lysosomal degradation of the core circadian clock protein brain and muscle ARNT-like 1 (BMAL1). Because BMAL1 transcriptionally activates the copper exporter ATP7A, its degradation reduces ATP7A expression, impairs copper efflux, and promotes further intracellular copper accumulation (Fig. 4). This self-amplifying interaction links circadian regulation to copper homeostasis. Nevertheless, the broader BMAL1–ATP7A regulatory axis promotes glioblastoma stem cell survival and tumor growth, partly through ATP7A-dependent regulation of fatty acid desaturation.^108^ These observations indicate that lysosomal degradation may either suppress or facilitate cuproptosis, depending on the protein target and cellular context.

The role of the lysosome extends beyond protein turnover to direct copper sequestration. Copper nanowires escape endolysosomal entrapment and efficiently induce cuproptosis, whereas copper nanoparticles retained within lysosomes fail to deliver a lethal copper load.^109^ The copper chelator tetrathiomolybdate attenuates periodontitis by inhibiting cuproptosis in macrophages, thereby restoring autophagy flux and preserving lysosomal integrity.^110^ Biomimetic nanozymes developed for osteoradionecrosis therapy similarly enhance mitophagy, as shown by increased mitochondrial-lysosomal colocalization.^111^ Preservation of lysosomal integrity and restoration of autophagic and mitophagic flux therefore counteract cuproptosis by maintaining mitochondrial health (Fig. 4).^111^

Collectively, these studies indicate that lysosomes regulate cuproptosis through at least three mechanisms: targeted degradation of copper transporters and regulatory proteins, sequestration of copper nanoparticles, and interaction with autophagic pathways that influence mitochondrial integrity. It remains unclear how these lysosomal functions are coordinated among different tissues and pathological conditions and whether therapeutic modulation of lysosomal activity can promote cuproptosis in cancer or suppress it in degenerative and inflammatory diseases. Future studies should define the molecular determinants of lysosomal cargo specificity and clarify the relationships among intralysosomal copper sequestration, autophagic degradation, and cellular copper homeostasis. Such information will be important for developing precision therapies targeting the lysosome–cuproptosis axis.

Lysosomes contribute to ferroptosis by regulating the labile iron pool through ferritinophagy, the selective autophagic degradation of the iron-storage protein ferritin (Fig. 4).^45^ Degradation of ferritin within lysosomes releases iron and increases cellular susceptibility to ferroptosis (Fig. 4). This process is tightly regulated. For example, the stimulator of interferon genes (STING)/NCOA4 axis promotes ferritinophagy and subsequent ferroptosis in acute kidney injury.^112^ Conversely, disruption of lysosomal function during cellular senescence, can impair ferritinophagy. This defect causes iron accumulation but paradoxically confers ferroptosis resistance on cancer cells.^113^ Therapeutic agents such as dihydroartemisinin can exploit this pathway by inducing lysosomal ferritin degradation, thereby increasing the labile iron pool and enhancing ferroptosis sensitivity in cancer cells.^114^

Apart from their role in iron storage and recycling, lysosomes have been identified as important initiation sites for ferroptosis through distinct but complementary mechanisms. One study showed that lipid peroxidation originates within lysosomes after GPX4 inhibition, using specialized probes that also function as potent ferroptosis inhibitors.^115^ Lysosomal lipid peroxidation induces lysosomal membrane permeabilization and triggers the release of Fe^2+^ (Fig. 4).^51,52^ The released iron then propagates lipid peroxidation to other organelles and promotes cell death. Another study supported this finding by demonstrating that the anti-ferroptotic compound liproxstatin-1 chelates redox-active iron within lysosomes.^116^ Building on this finding, the same group developed fentomycin-1, a bifunctional molecule that activates lysosomal iron to induce localized lipid peroxidation (Fig. 4).^116^ These findings highlight the therapeutic potential of targeting the lysosomal iron pool.

More recent studies show that copper also regulates ferroptosis through lysosomal pathways. Copper promotes ferroptosis by inducing autophagy-mediated degradation of GPX4, in lysosomes.^117^ In this context, copper chelators inhibit erastin-induced ferroptosis, whereas copper ions increase cellular susceptibility. Mechanistically, Cu^2+^ promotes GPX4 clustering and ubiquitination by selectively interacting with cysteine residues C107 and C148 (Fig. 4).^117^ This event activates TAX1-binding protein 1 (TAX1BP1)-dependent autophagy and results in lysosomal breakdown of GPX4. The loss of GPX4 increases lipid peroxidation and culminates in ferroptotic cell death (Fig. 4).^117^

These findings have also been validated in vivo. Copper enhanced the antitumor activity of erastin analogs in xenograft models,^118^ whereas copper chelation reduced ferroptosis-associated injury in psoriasis and atopic dermatitis.^119^ Taken together, these results identify the lysosome as a major regulatory site for ferroptosis initiation and propagation.

Even after lipid peroxidation damages the plasma membrane, cells can activate membrane repair machinery to delay cell death. During ferroptosis, plasma membrane injury produces a sustained increase in cytosolic Ca^2+^, which activates the endosomal sorting complex required for transport III (ESCRT-III) machinery. This is indicated by the formation of charged multivesicular body protein 4B (CHMP4B) puncta.^120^ This response is Ca^2+^ dependent because inhibition of Ca^2+^ influx by osmoprotectants prevents CHMP4B puncta formation.^120^ Functionally, ESCRT-III counteracts membrane damage and delays ferroptotic cell death, as depletion of the core components CHMP4B or CHMP4A increases cellular susceptibility to ferroptosis.^120,121^

Activation of ESCRT-III during ferroptosis also influences the inflammatory output of dying cells. Depletion of CHMP4B alters the cytokine secretion profile and enhances the capacity of supernatants from ferroptotic cells to activate macrophages.^120^ Ferroptotic stimulation also increases the expression of the ESCRT-III components CHMP4A and CHMP5, an effect reversed by the ferroptosis inhibitor ferrostatin-1. This upregulation is associated with increased release of small extracellular vesicles (sEVs). Knockdown of CHMP4A significantly reduces sEV production, indicating that ESCRT-III promotes sEV biogenesis under ferroptotic conditions.^121^ These sEVs exhibit an altered microRNA cargo, including reduced miR-433-3p. These microRNAs promote angiogenesis in recipient endothelial cells, thereby contributing to disease progression in experimental models of arthritis.^121^

#### Endoplasmic reticulum and peroxisomes in cuproptosis and ferroptosis regulation

The endoplasmic reticulum and peroxisomes are important sites in the regulation of cuproptosis and ferroptosis, integrating lipid metabolism, redox signaling, and stress responses that shape cell death sensitivity and execution.

The endoplasmic reticulum is a major source of cellular stress. The unfolded protein response is an endoplasmic reticulum stress signaling pathway. In lung adenocarcinoma, the unfolded protein response, particularly the spliced X-box binding protein 1 (XBP1s), functions as a tumor-protective mechanism to inhibit cuproptosis.^122^ Progressive copper accumulation correlates with unfolded protein response activation. During this response, XBP1s forms super-enhancers that alter mahogunin ring finger 1 (MGRN1) promoter-enhancer interactions, and promote MGRN1-mediated ubiquitination and degradation of lipoyltransferase 1 (LIPT1). This response suppresses cell death induced by copper-loaded ionophores such as elesclomol.^122^

The unfolded protein response also has complex links to ferroptosis. For instance, the unfolded protein response sensor inositol-requiring enzyme 1 alpha (IRE1α) regulates ferroptosis sensitivity by controlling the messenger RNA (mRNA) of GSH synthesis genes, glutamate-cysteine ligase catalytic subunit and SLC7A11. This activity occurs independently of its canonical unfolded protein response function.^123^

The endoplasmic reticulum can also be exploited to amplify cuproptosis in cancer therapy. A hollow calcium–copper bimetallic nanoplatform induces irreversible endoplasmic reticulum stress through ubiquitin–proteasome inhibition, calcium redistribution, and mitochondrial dysfunction. These coordinated effects activate a combined cuproptosis–paraptosis–apoptosis program that suppresses breast cancer growth.^124^ Similarly, the cuproptosis inducer YL21, when combined with copper ions, induces endoplasmic reticulum stress. This treatment triggers immunogenic cell death and activates antitumor immunity in murine breast cancer models. It also exhibits greater solubility and therapeutic efficacy than traditional disulfiram/Cu combinations.^125^

**Fig. 5:**
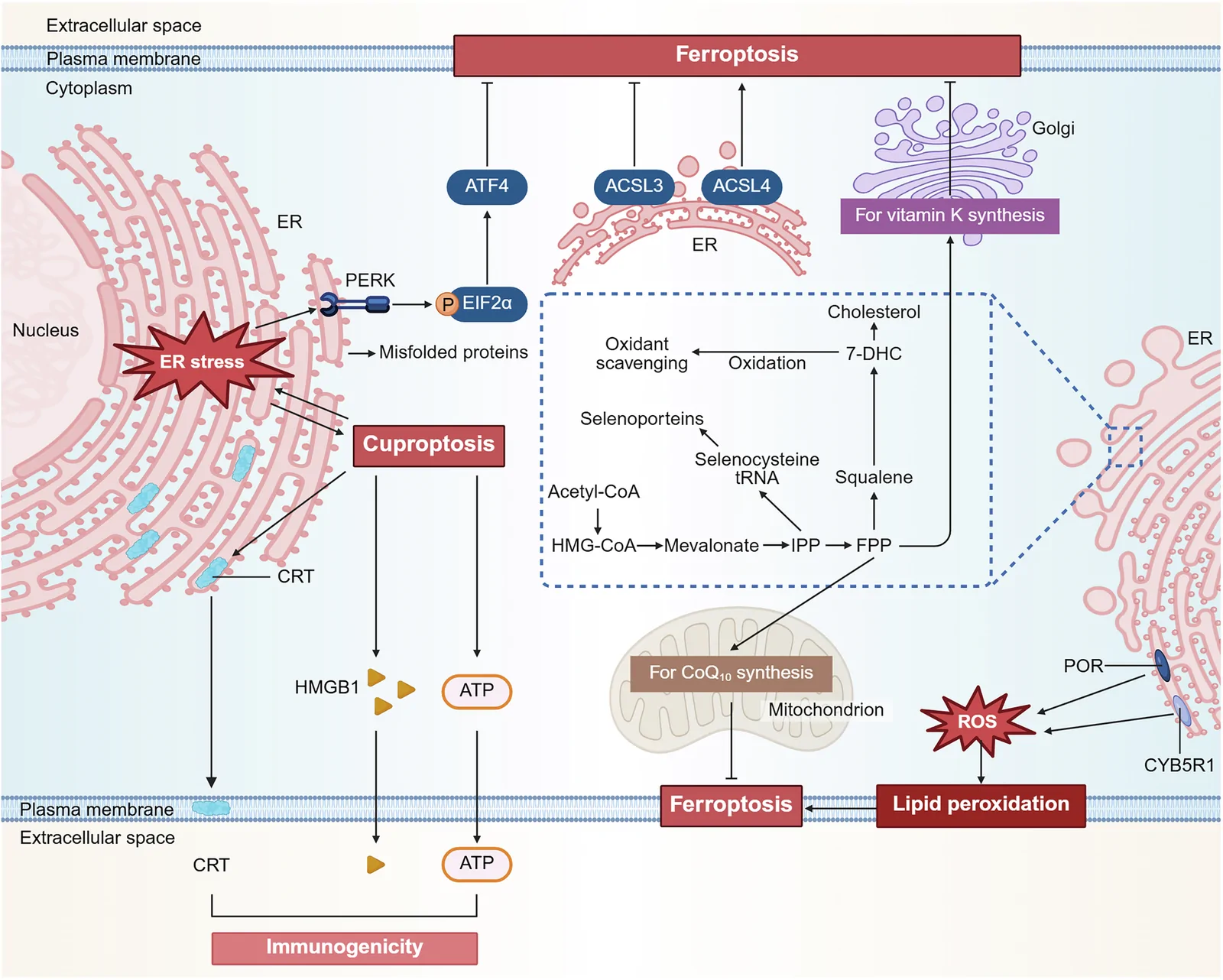
Roles of endoplasmic reticulum in cuproptosis and ferroptosis. 7-DHC 7-dehydrocholesterol, Acetyl-CoA acetyl-coenzyme A, CoQ_10_ coenzyme Q_10_, CRT calreticulin, ER endoplasmic reticulum, FPP farnesyl pyrophosphate, HMG-CoA hydroxymethylglutaryl-coenzyme A, IPP isopentenyl pyrophosphate. Created in BioRender. Li, Y. (2026) https://BioRender.com/pc22unu

Mechanistically, the endoplasmic reticulum provides an important link between cuproptosis and immune responses. In colorectal cancer, cuproptosis promotes reactive oxygen species (ROS) accumulation, which activates the protein kinase R-like endoplasmic reticulum kinase (PERK)–eukaryotic translation initiation factor 2 alpha (EIF2α) signaling axis and induces endoplasmic reticulum stress (Fig. 5).^126^ This response facilitates the accumulation of misfolded proteins and culminates in bona fide immunogenic cell death. A defining feature of this process is the translocation of calreticulin from the endoplasmic reticulum lumen to the plasma membrane, accompanied by the extracellular release of damage-associated molecular patterns, including high-mobility group box 1 (HMGB1) and ATP (Fig. 5).^126^ These signals promote antigen-presenting cell maturation and recruit cytotoxic T lymphocytes and M1 macrophages, thereby establishing an inflammatory tumor microenvironment. This immunogenic effect is further enhanced by in-situ cuproptosis-inducing systems, which strengthen immune cell activity and synergize with toll-like receptor 7 agonists such as imiquimod.^126^

The role of the endoplasmic reticulum in cuproptosis is further complicated by adaptive stress responses that confer resistance. In hepatocellular carcinoma, disulfiram/Cu induces endoplasmic reticulum stress together with both cuproptosis and ferroptosis. This stress activates the EIF2α-activating transcription factor 4 (ATF4) pathway, which protects cells against ferroptosis independently of xCT regulation. Disulfiram/Cu also causes compensatory accumulation of xCT by impairing its ubiquitin–proteasome-mediated degradation. Consequently, existing xCT protein persists longer and accumulates through a post-translational mechanism that is independent of ATF4 signaling (Fig. 5). This compensatory response restores GSH levels and reduces cellular susceptibility to ferroptosis, thereby creating an important mechanism of treatment resistance.^127^

Endoplasmic reticulum membrane-bound oxidoreductases such as cytochrome b5 reductase 1 (CYB5R1) and cytochrome P450 oxidoreductase (POR) utilize NADPH to generate ROS within the endoplasmic reticulum membrane and directly initiate lipid peroxidation (Fig. 5).^128^ The endoplasmic reticulum is well established as an initiation site for peroxidation. However, whether and how lipid peroxidation propagates from the endoplasmic reticulum to the plasma membrane to culminate in cell death remain unresolved. Proposed mechanisms involving vesicular trafficking or lipid transfer at membrane contact sites remain speculative and require direct experimental validation.^55^

As a major site of lipid metabolism, the endoplasmic reticulum contains numerous enzymes that shape cellular lipid composition and influence ferroptosis susceptibility. The balance between polyunsaturated fatty acids and monounsaturated fatty acids is particularly important, with a higher polyunsaturated fatty acid to monounsaturated fatty acid ratio favoring ferroptosis.^71,129^ Endoplasmic reticulum-resident fatty acid desaturases exert opposing effects on this balance.^130^ Calcium further modulates fatty acid desaturation through membrane-spanning 4-domains subfamily A member 15 (MS4A15).^131^ However, the physiological conditions under which this calcium-dependent regulation becomes rate-limiting for ferroptosis susceptibility remain poorly defined.

The acyl-coenzyme A synthetases ACSL4 and ACSL3 illustrate the complexity of endoplasmic reticulum-localized ferroptosis regulation (Fig. 5). These two synthetases are generally viewed as opposing regulators, with ACSL4 promoting polyunsaturated fatty acid incorporation and ferroptosis sensitivity, and ACSL3 favoring monounsaturated fatty acid incorporation and resistance. Exceptions nevertheless exist, such as the pro-ferroptotic role of ACSL3 in KRAS-mutant lung cancer.^66,72,76^ These findings indicate that simple binary models do not fully capture the context dependence of this pathway.

The mevalonate pathway in the endoplasmic reticulum also suppresses ferroptosis through multiple mechanisms. Within the mevalonate pathway, 3‑hydroxy‑3‑methylglutaryl‑coenzyme A synthase catalyzes the condensation of acetyl-coenzyme A with acetoacetyl-coenzyme A to form 3-hydroxy-3-methylglutaryl-coenzyme A (Fig. 5).^132^ This pathway subsequently generates isopentenyl pyrophosphate (Fig. 5),^133^ which is required for the isopenternylation of selenocysteine-tRNA, and thereby influences selenoprotein synthesis (Fig. 5).^89,134^ However, whether changes in this tRNA modification alter GPX4 abundance sufficiently to affect ferroptosis susceptibility awaits direct experimental verification. Seven-dehydrocholesterol functions as a sacrificial antioxidant (Fig. 5),^93^ whereas squalene confers protection through mechanisms that remain incompletely defined (Fig. 5).^135^ Farnesyl pyrophosphate acts as the essential precursor for mitochondrial coenzyme Q_10_ biosynthesis and vitamin K synthesis in the Golgi apparatus (Fig. 5).^136,137^

Peroxisomes have a dual role in cellular redox regulation because they contain both pro-oxidant enzymes such as xanthine dehydrogenase and nitric oxide synthase 2, and antioxidant enzymes such as SOD1.^138^ Recent findings indicate, however, that their contribution to ferroptosis is mediated primarily through lipid metabolism rather than from ROS or reactive nitrogen species.^68^ Peroxisomes initiate the synthesis of ether lipids, including the production of plasmalogen precursors. These lipids are further processed in the endoplasmic reticulum to generate polyunsaturated ether phospholipids, which sensitize cells to ferroptosis upon GPX4 inhibition.^68^ Interestingly, neurons deficient in plasmalogens exhibit increased vulnerability to oxidative damage, suggesting that these lipids may also function as endogenous antioxidants in certain contexts.^139^ In addition to lipid metabolism, peroxisomes contribute to steroid and peptide hormone biosynthesis, which may indirectly influence ferroptosis.^140^ Collectively, these findings indicate that peroxisomes regulate ferroptosis in a context-dependent manner, predominantly through ether lipid remodeling.

### Shared regulatory nodes and pathway crosstalk

Cuproptosis and ferroptosis are mechanistically distinct. Nevertheless, both mechanisms are closely linked to cellular metabolism and share several regulatory checkpoints. A major checkpoint is the balance between glycolysis and mitochondrial oxidative phosphorylation. The tumor suppressor p53 suppresses the Warburg effect and promotes mitochondrial respiration.^141^ The Warburg effect is a metabolic phenotype in which cells preferentially convert glucose to lactate through glycolysis even when oxygen is sufficient to support mitochondrial oxidative phosphorylation. Suppression of this phenotype has two important consequences. First, increased reliance on the TCA cycle enhances susceptibility to cuproptosis.^142^ Second, greater mitochondrial ROS production may promote ferroptosis by initiating lipid peroxidation.^143^ The mitophagy protein PTEN-induced kinase 1 (PINK1), for instance, suppresses colon tumor growth by promoting mitophagy and increasing mitochondrial respiration via activation of p53 signaling.^144^ This finding illustrates the close relationship among p53, mitochondrial function, and tumor suppression.

Glutathione also has context-dependent roles in cuproptosis and ferroptosis through its functions in metal chelation and redox homeostasis. Glutathione acts as a major inhibitor of ferroptosis. The xCT–GSH–GPX4 axis provides a powerful defense against ferroptotic cell death.^145^ Depletion of GSH through consumption, impaired synthesis, or oxidation compromises GPX4 activity.^145^ This inactivation disrupts cellular antioxidant defense, and results in the accumulation of ROS and lipid peroxides, ultimately triggering ferroptosis.^145^ In cuproptosis, GSH acts through a different mechanism by chelating copper ions and limiting the labile intracellular copper pool. Depletion of GSH removes this buffering capacity and increases cellular sensitivity to cuproptosis induced by agents such as disulfiram/Cu.^145^ This sensitization is associated with ROS accumulation and aggregation of lipoylated DLAT, both of which are hallmarks of cuproptosis.^145^

The hypoxic tumor microenvironment also exerts a strong influence on cellular metabolism and cell death.^146^ Hypoxia-inducible factors (HIFs) are master regulators of the hypoxic response.^147^ Recent work shows that HIF-1α promotes resistance to cuproptosis by activating pyruvate dehydrogenase kinase (PDK) 1 and 3 (which reduces the level of the copper target DLAT), and by promoting the expression of copper-sequestering metallothioneins.^148^ Elevated copper concentrations can, in turn, stabilize HIF-1α and establish a feedback loop.^148^

The HIFs also exert context-dependent effects on ferroptosis. In human non-small cell lung cancer, fibrosarcoma, and acute promyelocytic leukemia cell lines, HIF-1α suppresses ferroptosis by promoting lipid storage.^149^ Conversely, in renal cell carcinoma-derived cells, HIF-2α promotes ferroptosis through transcriptional upregulation of hypoxia-inducible, lipid droplet-associated protein. This response increases polyunsaturated fatty acid production and subsequent lipid peroxidation.^150^ The context-dependent functions of HIF signaling suggest that selecting of patients for HIF-targeted therapy may benefit from assessment of ferroptosis-regulating gene expression in tumor cells.^151^

Taken together, these interactions indicate that the cellular responses to metal stress do not reflect a simple choice between cuproptosis and ferroptosis. Instead, cell fate is determined by the integrated effects of metabolic state, microenvironmental conditions, and the activity of major signaling hubs.

### Multilayered regulatory networks

**Fig. 6:**
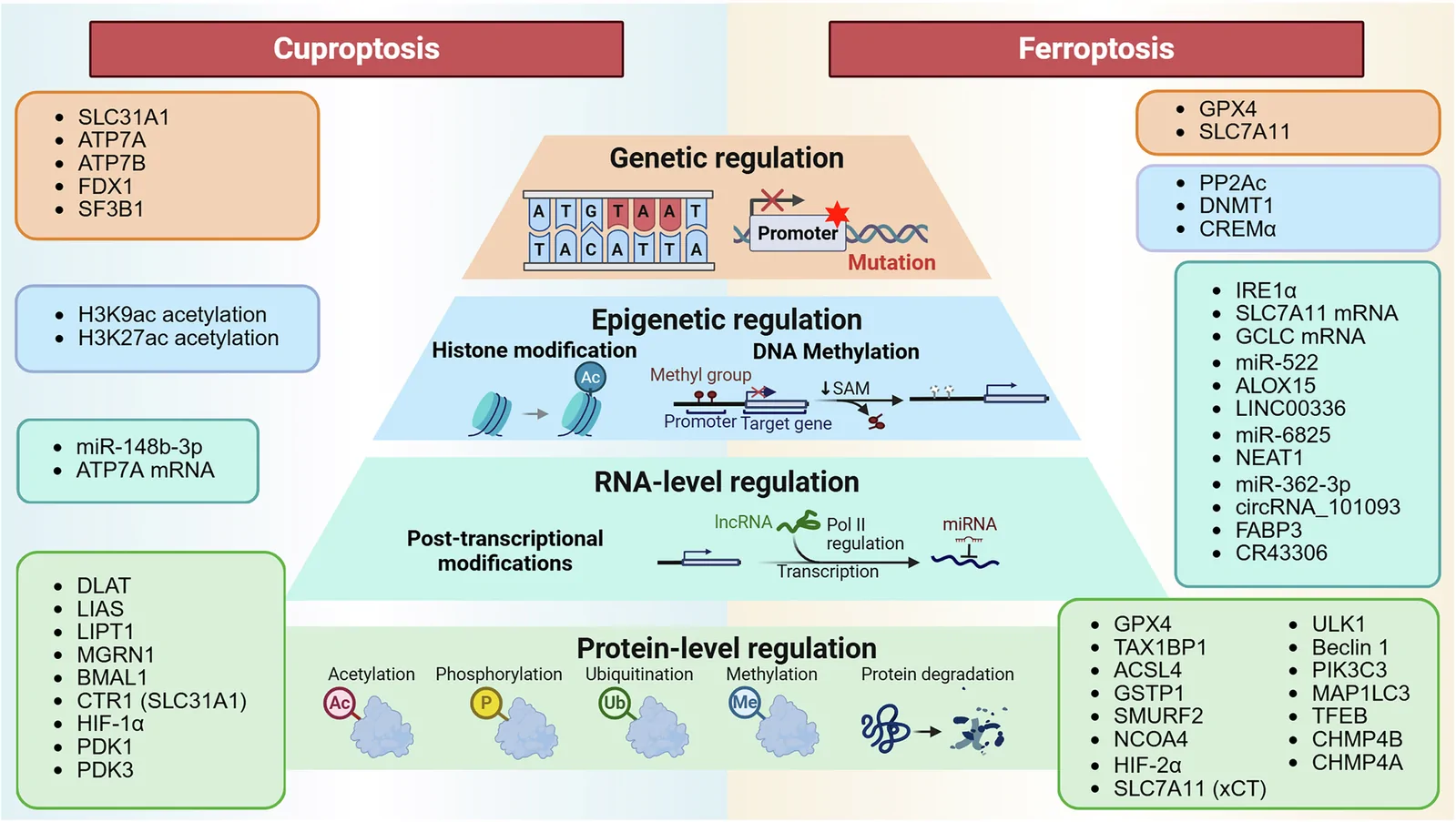
Multi-layered regulatory network of cuproptosis and ferroptosis. This schematic summarizes representative molecules that regulate cuproptosis and ferroptosis at genetic, epigenetic, transcriptional, post-transcriptional, and post-translational levels. The left panel shows regulatory mechanisms involved in cuproptosis. The right panel shows regulatory mechanisms involved in ferroptosis. ALOX15 arachidonate lipoxygenase 15, GCLC glutamate-cysteine ligase catalytic subunit, Pol II RNA polymerase II, SAM S-adenosylmethionine, TFEB transcription factor EB. Created in BioRender. Li, Y. (2026) https://BioRender.com/pc22unu

Cellular susceptibility to cuproptosis and ferroptosis is not fixed. Instead, it is dynamically regulated at the genetic, epigenetic, transcriptional, post-transcriptional, and post-translational levels (Fig. 6). These interconnected regulatory layers establish baseline susceptibility and enable rapid adaptation to changing cellular and environmental conditions.

#### Genetic and epigenetic regulation of baseline sensitivity

The genetic background of a cell is a major determinant of its susceptibility to metal-dependent cell death. Mutations in genes encoding key regulators can markedly alter this sensitivity. In cuproptosis, genetic variation in copper transporters such as SLC31A1, ATP7A, and ATP7B, or in regulators such as FDX1, would be expected to influence cellular susceptibility.^42^ A notable example involves acute myeloid leukemia cells carrying mutations in the splicing factor 3b subunit 1 (SF3B1). These mutations cause aberrant splicing and reduced expression of ATP-binding cassette subfamily B member 7 (ABCB7), a mitochondrial Fe–S cluster transporter.^152^ The resulting impairment of Fe–S cluster biogenesis creates a specific vulnerability to copper ionophore-induced cuproptosis.^152^

The eukaryotic H3-H4 histone tetramer has been identified as a cupric reductase that catalyzes the reduction of Cu^2+^ to Cu^+^ at the H3-H3’ interface via the conserved residues C110 and H113.^153^ In yeast, mutations at H113 (e.g. H113N) impair this reductase activity, diminish intracellular Cu^+^ availability, and compromise the function of copper-dependent enzymes such as cytochrome c oxidase and SOD1. These effects increase cellular sensitivity to copper restriction.^153^ Conversely, the gain-of-function A110C mutation enhances cupric reductase activity, improves Cu^+^ availability, and confers resistance to copper limitation.^153^ These findings indicate that histone mutations can directly alter cellular copper metabolism and may influence susceptibility to cuproptosis.

Mutations in GPX4 or SLC7A11 can likewise increase cellular sensitivity to ferroptosis.^70^ A missense mutation in GPX4 (p.R152H) was recently identified in a patient with early-onset neurodegeneration. This variant disrupted membrane anchoring without markedly impairing catalytic activity. Neuron-specific expression of GPX4 p.R152H in mice caused ferroptosis-dependent neuronal loss. These findings provide direct evidence that ferroptosis contributes to neurodegeneration.^154^

Apart from the fixed genetic variation, epigenetic modifications provide a dynamic layer of gene regulation.^155–157^ Through histone modification and DNA methylation, cells can remodel chromatin and alter their susceptibility to cuproptosis and ferroptosis.^158^ These changes do not alter the underlying DNA sequence. However, they may be influenced by environmental factors and contribute to the pathogenesis of cancer, cardiovascular disease, and autoimmune disorders.^159–161^

In Wilson’s disease, copper overload increases acetylation of histone 3 lysine 9 (H3K9) and histone 3 lysine 27 (H3K27). This observation indicates that histone modifications function as downstream epigenetic mediators of copper accumulation.^162^ Epigenetic mechanisms, including DNA methylation, also regulate ferroptosis by altering the expression of ferroptosis-related proteins.^161^ Protein phosphatase 2 A catalytic subunit (PP2Ac) promotes DNA hypomethylation by reducing DNA methyltransferase 1 (DNMT1) expression and inducing demethylation of specific gene promoters.^160^ This hypomethylation response increases the expression of transcriptional repressors such as cyclic adenosine monophosphate–responsive element modulator alpha (CREMα), which binds to the promoter of GPX4 and suppresses its transcription.^160^ Reduced GPX4 expression increases lipid ROS and induces ferroptosis in neutrophils. These ferroptotic neutrophils release autoantigens that activate autoreactive B cells and plasmacytoid dendritic cells, thereby sustaining a pathogenic feedback loop in systemic lupus erythematosus.^163^

#### RNA-level orchestration: post-transcriptional control

Regulation at the RNA level adds another layer of complexity to cuproptosis and ferroptosis and enables rapid adaptation to changing cellular conditions. This regulation includes non-coding RNAs and post-transcriptional modifications that fine-tune gene expression.

MicroRNAs are small non-coding RNAs that regulate gene expression post-transcriptionally by binding to the 3’ untranslated region of target mRNAs, thereby promoting their degradation or inhibiting translation.^164^ In oral squamous cell carcinoma, exosomes derived from cancer-associated fibroblasts contain reduced levels of miR-148b-3p.^165^ Loss of this microRNA in recipient tumor cells relieves post-transcriptional repression of ATP7A. Functional assays confirmed that miR-148b-3p binds directly to the 3’ untranslated region of ATP7A mRNA and suppresses ATP7A expression. Consequently, reduced miR-148b-3p expression increases ATP7A abundance and accelerates copper efflux. This response suppresses cuproptosis, as shown by reduced FDX1, lipoic acid synthase (LIAS), and decreased protein lipoylation.^165^ Conversely, restoration of miR-148b-3p or knockdown of ATP7A enhances cuproptosis and attenuates malignant behavior both in vitro and in vivo.^165^

Exosomal miR-522 secreted by cancer-associated fibroblasts suppresses ferroptosis by targeting arachidonate lipoxygenase 15, thereby promoting chemoresistance in gastrointestinal cancer.^166^ Similarly, long intergenic non-protein coding RNA 336 (LINC00336) acts as a competitive endogenous RNA by sequestering miR-6825. This interaction increases cystathionine-β-synthase expression, suppresses ferroptosis, and promotes lung cancer cell proliferation.^167^ Conversely, nuclear paraspeckle assembly transcript 1 (NEAT1), another long non-coding RNA, enhances ferroptosis by regulating the miR-362-3p/myo-inositol oxygenase axis. These observations illustrate the bidirectional regulatory effects of non-coding RNAs on ferroptosis and their capacity to alter tumor-cell sensitivity to therapy.^168^ The long non-coding RNA CR43306 has also been implicated in testicular aging in *Drosophila*, a process exacerbated by copper overload and associated with ferroptosis. This finding further links non-coding RNA regulation with metal stress and cell death.^169^

Plasma exosomes from patients with lung adenocarcinoma reduce ferroptosis sensitivity in cancer cells.^170^ This effect was mediated by the exosomal circular RNA circRNA_101093. After entering recipient cells, circRNA_101093 increases the abundance of fatty acid-binding protein 3 (FABP3).^170^ The FABP3, in turn, facilitates the conversion of arachidonic acid, a major substrate for lipid peroxidation, into N-arachidonoyl taurine.^170^ This metabolic shift reduces the availability of arachidonic acid for incorporation into the plasma membrane phospholipids and protects the cell from ferroptotic damage.^170^ This study provides a clear example of how an exosome-delivered circular RNA can reprogram lipid metabolism and confer ferroptosis resistance.

#### Protein-level regulation: post-translational modifications and proteostasis

The final regulation and execution of cuproptosis and ferroptosis occur at the protein level. Post-translational modifications, protein folding, and degradation systems determine the activity, localization, and abundance of effector and regulatory proteins.

Post-translational modifications include phosphorylation, ubiquitination, glycosylation, palmitoylation, lactylation, acetylation, and other covalent protein modifications.^171^ Lipoylation is central to cuproptosis. The attachment of lipoic acid to mitochondrial enzymes such as DLAT creates the molecular targets for copper-induced toxicity. Accordingly, the enzymes involved in protein lipoylation, including LIAS and LIPT1, act as positive regulators of cuproptosis.^99^

Acetylation also regulates autophagy, which is closely linked to ferroptosis. Components of the autophagy machinery, including unc-51 like autophagy activating kinase 1 (ULK1) and beclin 1-phosphatidylinositol 3-kinase catalytic subunit type 3 (PIK3C3) initiation complexes, the microtubule associated protein 1 light chain 3 (MAP1LC3) lipidation system, and the master transcriptional regulator transcription factor EB, are all targets of acetylation.^172^ This modification regulates their activity, molecular interactions, and subcellular localization, thereby influencing autophagy from initiation to cargo recognition and lysosomal fusion.^172^ Altered glycosylation, a hallmark of cancer, also contributes to metastasis, immune evasion, and abnormal ferroptotic responses. Changes in the glycosylation of cell-surface receptors or metabolic enzymes may alter their activity and stability, thereby modifying cellular susceptibility to ferroptosis.^173^

Protosappanin A protects against doxorubicin-induced cardiotoxicity by binding directly to ACSL4 and inhibiting its phosphorylation, thereby suppressing phospholipid peroxidation and ferroptosis.^174^ Ubiquitination, the attachment of ubiquitin chains to a target protein, is best known for tagging proteins for degradation by the proteasome. Nevertheless, it also participates in signaling and protein localization.^94,117^ Exogenous copper increases GPX4 ubiquitination and promotes the formation of GPX4 aggregates. These aggregates are recognized by the autophagy receptor TAX1-binding protein-1 for lysosomal degradation.^117^ Removal of GPX4 weakens a major cellular defense against lipid peroxidation and promotes ferroptotic death.^117^ The SMAD-specific E3 ubiquitin protein ligase 2 (SMURF2) also promotes ubiquitination and degradation of GSTP1, a GPX4-independent ferroptosis suppressor. This degradation sensitizes cancer cells to ferroptosis.^94^

Collectively, these regulatory layers ensure that commitment to cuproptosis or ferroptosis reflects an integrated response to multiple intracellular and extracellular signals.

### Crosstalk with other cell death pathways and immune responses

Cuproptosis and ferroptosis do not occur in isolation. Instead, they interact extensively with other forms of RCD and with the immune signaling to create an interconnected cell death network.

Substantial crosstalk exists between metal-dependent cell death and apoptosis. Copper ions can induce apoptosis at specific concentrations or in particular cell types, often through mitochondrial pathways involving ROS generation and cytochrome c release.^41^ The tumor suppressor p53, a classical inducer of apoptosis, can also promote ferroptosis, suggesting it functions as a shared regulatory node among different death programs.^175^ In some settings, resistance to apoptosis increases cancer cell susceptibility to ferroptosis. This phenomenon, known as “phenotype switching,” has important therapeutic implications.^176^ Necrostatin-1, a receptor interacting protein kinase 1 inhibitor used to study necroptosis, was subsequently shown to inhibit ferroptosis, further illustrating the mechanistic overlap among these pathways.^1^ Coactivation of necroptosis and ferroptosis has also been reported in pathological conditions such as kidney disease, in which both pathways contribute to tubular injury.^1^

Autophagy is another major regulator of cuproptosis and ferroptosis. In cuproptosis, protective autophagy may be activated as a compensatory response to copper overload.^177^ Pharmacological inhibition of autophagy with chloroquine, or genetic knockout of essential autophagy genes increases the susceptibility of cancer cells to cuproptosis. In contrast, autophagy induction with rapamycin attenuates cuproptosis.^177^ Autophagy can also promote ferroptosis by degrading protective proteins such as GPX4 or by increasing iron availability through ferritinophagy.^41,117,178^ In other contexts, however, autophagy protects cells by removing damaged organelles and proteins.^178^ The role of autophagy in cancer is therefore context dependent. During early tumor development, autophagy may suppress tumorigenesis, whereas in established tumors it may promote survival and treatment resistance. These effects are mediated partly through modulation of susceptibility to ferroptosis and related cell death pathways.^179^

An important aspect of RCD is its immunogenicity. Apoptosis is generally regarded as immunologically silent, whereas necrotic forms of RCD, including cuproptosis and ferroptosis, are often immunogenic because they release damage-associated molecular patterns such as HMGB1 and ATP. These signals promote inflammation and recruit immune cells to the sites of tissue injury.^3^ Induction of immunogenic cell death is therefore a major objective in cancer therapy. Cuproptosis and ferroptosis can enhance dendritic cell-mediated antigen presentation and promote the infiltration and activation of cytotoxic T lymphocytes.^118,180^ These properties make both pathways attractive targets for combination with immunotherapies, including immune checkpoint blockade.

## Pathophysiological roles and disease implications

Dysregulation of cuproptosis and ferroptosis has pathogenic consequences that extend well beyond the cellular level. Inappropriate activation or suppression of these metal-dependent cell death pathways contributes to the onset, progression, and therapeutic response of diverse human diseases, ranging from acute tissue injury and chronic degenerative disorders to cancer.

### Cardiovascular diseases

Dysregulation of copper and iron is increasingly recognized as a contributor to cardiomyocyte vulnerability during ischemic injury, cardiac remodeling, and heart failure.^181^ Copper homeostasis is also closely associated with atherosclerosis, and several cuproptosis-related genes have been implicated in its pathogenesis. Integrated analysis of bulk and single-cell sequencing data from atherosclerotic plaques showed increased expression of FDX1 and the copper importer SLC31A1, together with reduced expression of glutaminase, an enzyme involved in glutaminolysis.^182^ These genes formed a cuproptosis-related diagnostic signature for atherosclerosis. Specifically, SLC31A1 was localized predominantly to macrophages within atherosclerotic lesions. This finding suggests that copper uptake and subsequent cuproptosis in these immune cells may contribute to plaque progression.^182^

Ferroptosis contributes substantially to cardiomyocyte loss in ischemic heart disease and heart failure.^183^ Myocardial ischemia–reperfusion injury, a paradoxical consequence of restoring blood flow to ischemic tissue, remains a major clinical challenge in which ferroptosis has an important role.^184^ During reperfusion, a burst of ROS production, combined with iron dysregulation, creates a strongly pro-ferroptotic environment. Apoptosis and necrosis appear to dominate during the early phase of ischemia–reperfusion injury, whereas ferroptosis becomes increasingly prominent during prolonged reperfusion.^185^

Several molecular pathways contribute to ischemia–reperfusion-induced ferroptosis. The STING signaling pathway, traditionally associated with innate immunity, aggravates myocardial injury by promoting autophagic degradation of GPX4. The resulting STING–GPX4 feedback loop amplifies cardiomyocyte ferroptosis. Genetic deletion of STING or pharmacological inhibition with H-151 reduces ischemia–reperfusion injury.^186^ Arachidonate lipoxygenase 15 is another important regulator. Its metabolite, 15-hydroperoxyeicosatetraenoic acid (15-HpETE), accumulates in ferroptotic cardiomyocytes. The 15-HpETE triggers ferroptosis by promoting the ubiquitin-dependent degradation of peroxisome proliferator-activated receptor gamma coactivator 1-alpha (Pgc1α), an important regulator of mitochondrial biogenesis. Pharmacological inhibition of arachidonate lipoxygenase 15 with ML351 protects the myocardium and restores cardiac function in a murine ischemia–reperfusion model.^185^ In addition, toxic aldehydes such as 4-hydroxy-2-nonenal generated during ischemia–reperfusion can trigger GPX4 degradation. Ovarian tumor deubiquitinase 5 has been identified as a novel protector that stabilizes GPX4, and its elevation can reverse 4-hydroxy-2-nonenal-induced ferroptosis and mitigate ischemia–reperfusion injury.^187^

Ferroptosis also contributes to pathological cardiac remodeling in heart failure. Expression of ACSL4, an enzyme that enriches membranes with peroxidation-susceptible polyunsaturated fatty acids, is markedly increased in pressure overload-induced heart failure. Activation of the pyroptotic signaling pathway by ACSL4-dependent ferroptosis results in the production of the proinflammatory cytokine interleukin-1β and exacerbation of cardiac dysfunction. Pharmacological inhibition or genetic deletion of *ACSL4* improves cardiac function in murine heart failure models, identifying the ferroptosis-pyroptosis cascade as a therapeutic target.^188^

Ferroptosis is also implicated in chemotherapy-induced cardiotoxicity. Doxorubicin, a potent anticancer agent, induces cardiomyocyte ferroptosis by promoting ACSL4 phosphorylation and autophagic degradation of ferritin heavy chain 1 (FTH1), resulting in lipid peroxidation and Fe^2+^ release. Cellular repressor of E1A-stimulated genes (CREG1) alleviates doxorubicin-induced cardiotoxicity by suppressing cardiomyocyte ferroptosis. Inhibition potentially occurs through the F-box and WD repeat domain containing 7 (FBXW7)/forkhead box O1 (FOXO1)/PDK4 pathway.^189^

The gut microbiome has also emerged as a regulator of cardiac ferroptosis. In age-related heart failure, the abundance of the gut bacterium *Faecalibacterium prausnitzii* and its metabolite butyrate is reduced. Oral administration of *F. prausnitzii* or butyrate attenuated age-related heart failure by suppressing cardiomyocyte ferroptosis. The mechanism involves downregulation of lipocalin 2 and subsequent reduction of intracellular iron accumulation.^190^

Recognition of cuproptosis and ferroptosis as contributors to cardiovascular disease has created new therapeutic opportunities. Targeting these pathways may reduce cardiomyocyte death, limit inflammation, and improve cardiac function after injury.^181^ Therapeutic manipulation of cuproptosis is an emerging concept. Nanozymes designed to induce cuproptosis and ferroptosis selectively in senescent cells within atherosclerotic plaques represent one innovative approach.^191^ Because both pathways are closely linked to mitochondrial metabolism, interventions that restore mitochondrial health may also suppress pathological metal-dependent cell death.^192^

Several ferroptosis inhibitors and modulators have shown promise in preclinical studies. Hydrogen sulfide protects cardiomyocytes from doxorubicin-induced ferroptosis by activating the nuclear factor erythroid 2-related factor 2 (Nrf2)-dependent SLC7A11–GSH–GPX4 antioxidant pathway.^193^ The arachidonate lipoxygenase 15 inhibitor ML351 prevents 15-HpETE-mediated ferroptosis and improves cardiac function after ischemia–reperfusion injury.^185^ Similarly, inhibiting STING with H-151 stabilizes GPX4 and alleviates ischemia–reperfusion injury.^186^

Repurposing existing drugs represents another promising strategy. Dexmedetomidine ameliorates ischemia–reperfusion injury by regulating metabolic reprogramming, reducing malate dehydrogenase 2 lactylation, and suppressing ferroptosis.^194^ Empagliflozin, a sodium–glucose cotransporter 2 inhibitor used to treat diabetes and heart failure, reduces ferroptosis, fibrosis, and inflammation in a nondiabetic murine model of doxorubicin-induced cardiotoxicity. This finding suggests that ferroptosis inhibition may contribute to its cardioprotective effects.^195^ Non-coding RNAs are also emerging as potential therapeutic targets. A ferroptosis-associated circular RNA suppresses cardiomyocyte ferroptosis by stabilizing nicotinamide phosphoribosyltransferase.^196^ This enzyme in turn activates the sirtuin1/FOXO1/FTH1 signaling axis and protects the heart from ischemia–reperfusion injury.^196^

Clinical translation will require careful consideration of systemic iron metabolism. Iron deficiency is common in heart failure and is associated with worse symptoms and clinical outcomes.^197^ Systemic iron chelation may therefore be detrimental. Therapeutic strategies should selectively suppress pathological iron-dependent lipid peroxidation within injured cardiac tissue without disrupting physiological iron homeostasis.

### Neurodegenerative diseases

Neurodegenerative diseases such as Alzheimer’s disease, Parkinson’s disease, Huntington’s disease, and amyotrophic lateral sclerosis are characterized by the progressive loss of specific neuronal populations. Dysregulation of metal homeostasis, particularly the accumulation of copper and iron in the brain, is a well-documented feature of these disorders. Metal-dependent cell death may therefore have an important role in their pathogenesis.^7^

Copper dysregulation is strongly implicated in neurodegenerative diseases.^7^ The discovery of cuproptosis has provided a new framework for understanding the neurotoxic effects of copper overload.^34^ Copper dysregulation is a well-documented in Alzheimer’s disease. A direct mechanistic link between amyloid beta pathology and cuproptosis was recently demonstrated using fluorescent probes capable of simultaneously visualizing Cu^+^ and Cu^2+^ in living neurons.^198^ Amyloid beta oligomerization promoted intracellular copper accumulation, with elevated Cu^+^ levels inducing ROS generation, aggregation of lipoylated proteins, and FDX1-dependent cuproptosis.^198^ Chelation of Cu^+^ or knockdown of FDX1 completely prevented this form of cell death. This observation establishes cuproptosis as the major downstream effector of amyloid beta-induced neurotoxicity.^198^ These findings shift the interpretation of copper toxicity from a nonspecific oxidative stress mechanism toward a defined form of RCD.

Ferroptosis is increasingly recognized as an important contributor to the pathogenesis of Alzheimer’s disease and Parkinson’s disease.^199^ Recent studies have begun to unravel the specific molecular mechanisms linking Alzheimer’s disease pathology to ferroptosis. In a mouse model of Alzheimer’s disease, treatment with a bacterium or its metabolite lysophosphatidylcholine reduced amyloid beta accumulation and improved cognitive function. Mechanistically, lysophosphatidylcholine activates G protein-coupled receptor 119 (GPR119), suppresses ACSL4 expression, thereby suppressing ferroptosis and alleviating Alzheimer’s disease-related pathology.^200^ Other therapeutic strategies target the central ferroptosis machinery more directly. Forsythoside A mitigates Alzheimer’s disease-like pathology by activating the Nrf2/GPX4 axis, thereby strengthening antioxidant defense against lipid peroxidation.^201^ Thonningianin A has similarly been identified as a ferroptosis inhibitor that directly binds to and activates GPX4, ameliorating Alzheimer’s disease pathology in both cellular and animal models.^202^

In Parkinson’s disease, the hallmark pathological feature is the progressive loss of dopaminergic neurons in the substantia nigra, a region in which iron accumulates with aging. This iron accumulation, together with oxidative stress and mitochondrial dysfunction, creates conditions favorable for ferroptosis.^203,204^ Aggregation of α-synuclein, a central event in Parkinson’s disease, generates ROS and lipid peroxides, and may thereby trigger ferroptosis.^204^ Glial cells, particularly microglia and astrocytes, are important participants in this process. Activated glia can create an iron-rich, proinflammatory microenvironment that promotes ferroptosis in adjacent neurons, highlighting the importance of glia–neuron crosstalk in Parkinson’s disease neurodegeneration.^205^ A further link between lysosomal dysfunction and ferroptosis has also been identified. The cystine/glutamate antiporter SLC7A11 functions as an unconventional proton transporter in lysosomes. Its inhibition causes lysosomal over-acidification, impaired degradation, and ferroptosis, and facilitates α-synuclein aggregation in neurons.^206^ This finding directly links a core component of the ferroptosis pathway with lysosomal homeostasis and Parkinson’s disease pathology.

Copper and iron have attracted attention as therapeutic agents because of strong preclinical evidence linking metal dyshomeostasis to neurodegeneration. Metal chelators, which bind and remove excess metal ions, have been evaluated in clinical trials for both Alzheimer’s disease and Parkinson’s disease. Results have generally been disappointing and in some cases harmful, prompting reassessment of this strategy.

A phase II randomized clinical trial examining the brain-permeable iron chelator deferiprone in early Alzheimer’s disease showed that despite reducing hippocampal iron levels, treatment was associated with faster cognitive decline than placebo.^207^ This worsening was caused largely by poorer performance on executive function tests and was accompanied by increased volume loss in frontal brain regions.^207^ Similarly, a phase II trial of deferiprone in patients with newly diagnosed Parkinson’s disease also produced unfavorable results. Over 36 weeks, participants receiving deferiprone had significantly worse parkinsonism scores than those receiving placebo. A large proportion of the participating subjects required initiation of dopaminergic therapy because of symptom progression.^208^

These clinical failures contrast sharply with encouraging preclinical findings and illustrate the complex role of metals in the brain. A recent mechanistic framework may help explain this paradox. This framework proposes that apparent iron overload in neurodegenerative diseases may reflect functional iron deficiency and disrupted iron homeostasis, rather than excess redox-active labile iron.^209^ In disease states marked by mitochondrial dysfunction and bioenergetic insufficiency, iron chelation may restrict the iron required for mitochondrial ATP production. This may compromise ATP-dependent GSH synthesis and weaken ferroptosis defenses.^202^ This framework provides a plausible explanation for why nonselective iron removal accelerated cognitive and motor decline in deferiprone trials.^209^

Excess iron can be toxic, yet iron is also indispensable for numerous physiological processes, and aggressive non-selective chelation may disrupt these functions and produce adverse consequences. These results suggest that simply lowering total iron content is unlikely to be beneficial and may even be harmful. Future strategies will probably need greater precision, perhaps by redistributing iron within cells, limiting iron-catalyzed oxidative reactions without removing the metal itself, or targeting the specific pathological iron pools involved in ferroptosis.

Copper chelation therapy is the standard of care for Wilson’s disease, a genetic disorder of copper overload. Drugs such as tetrathiomolybdate and trientine effectively reduce copper burden and control disease manifestations.^210,211^ However, Wilson’s disease is a monogenic disorder with a clear and primary cause, namely copper accumulation. This feature differs fundamentally from multifactorial neurodegenerative disorders such as Alzheimer’s disease and Parkinson’s disease in which metal dyshomeostasis is only one of several interacting pathological processes. The success in Wilson’s disease therefore cannot be directly extrapolated to neurodegenerative conditions. The negative deferiprone trials emphasize the need for deeper understanding of the disease-specific roles of metal-dependent death pathways and for development of more selective interventions that can modulate pathological mechanisms without disrupting essential physiological functions.

### Autoimmune and inflammatory diseases

The interplay between metal-dependent cell death and the immune system is an expanding area of investigation with important implications for autoimmune and inflammatory diseases. Cuproptosis and ferroptosis are increasingly recognized not merely as consequences of tissue injury but as active mediators of inflammation and immune dysregulation. In systemic lupus erythematosus and rheumatoid arthritis, these pathways contribute to self-perpetuating cycles of cell death, damage-associated molecular pattern release, and sustained immune activation, thereby promoting chronic inflammation and progressive tissue destruction.^212,213^

Systemic lupus erythematosus is a systemic autoimmune disease characterized by autoantibody production and widespread inflammation. Neutrophil ferroptosis has been identified as an important pathogenic event.^163,214^ Neutrophils from both lupus-prone mice and patients with systemic lupus erythematosus show increased susceptibility to ferroptosis. This heightened vulnerability is promoted by the inflammatory milieu of systemic lupus erythematosus, in which autoantibodies and interferon-α lead to suppressed expression of the ferroptosis regulator GPX4.^163,214^ The resulting neutrophil ferroptosis contributes directly to neutropenia, a common clinical feature of systemic lupus erythematosus. The release of pro-inflammatory mediators from dying neutrophils further fuels systemic autoimmunity. Treatment with a specific ferroptosis inhibitor significantly reduced disease severity in lupus-prone mice. This finding established a causal link between neutrophil ferroptosis and lupus pathogenesis.^163,214^

In rheumatoid arthritis, synovial fibroblasts drive joint destruction by forming pannus that invades cartilage and bone.^215^ These fibroblasts are susceptible to ferroptosis induced by agents like imidazole ketone erastin. However, their sensitivity is modified by the inflammatory cytokine tumor necrosis factor (TNF), which promotes GSH biosynthesis that protects the fibroblasts against ferroptosis.^216^ Consequently, combining a TNF antagonist with a low-dose ferroptosis inducer synergistically triggers ferroptotic cell death in synovial fibroblasts and attenuates arthritis progression in a murine rheumatoid arthritis model.^216^

The role of macrophages in rheumatoid arthritis-associated ferroptosis is also complex and context-dependent. Studies have shown that macrophage subtypes differ in their susceptibility to this pathway. Anti-inflammatory M2 macrophages are highly vulnerable to iron-induced ferroptosis, whereas pro-inflammatory M1 macrophages are more resistant, partly because of differences in GPX4 degradation pathways.^217,218^ This differential susceptibility disturbs the M1/M2 balance and shifts the microenvironment toward a more pro-inflammatory state. During ferroptosis, dying M2 macrophages release HMGB1, which activates toll-like receptor 4 signaling in M1 macrophages, further amplifying the inflammatory response. Inhibiting ferroptosis with agents such as liproxstatin-1 rescues M2 macrophages and alleviates arthritis.^217,218^ Ferroptosis of articular chondrocytes also contributes to joint destruction in rheumatoid arthritis.^219^ This process is mediated by the transient receptor potential melastatin 7 (TRPM7) channel. When activated, this channel causes calcium influx and activation of the protein kinase C alpha (PKCα)/NADPH oxidase 4 (NOX4) axis, culminating in oxidative stress and ferroptosis. Inhibition of TRPM7 has been shown to protect chondrocytes and alleviate cartilage damage.^219^

Cuproptosis is also being investigated as a regulator of antitumor immunity because of its capacity to induce immunogenic cell death. An intelligent nanosystem designed to release copper ions and the copper ionophore elesclomol within tumor cells induced cuproptosis and simultaneously remodeled the immunosuppressive tumor microenvironment.^220^ This response increased tumor-infiltrating lymphocytes and inflammatory cytokine secretion, thereby enhancing the efficacy of immune checkpoint blockade.^220^ These findings suggest that cuproptosis can convert an immunologically “cold” tumor into a more immunologically active tumor.

Studies of bacterial infection provide additional insight into the immunomodulatory effects associated with cuproptosis-related processes. A nanoreactor designed to induce cuproptosis-like death in bacteria within biofilms also reactivated macrophages. Its chemodynamic therapy component enhanced macrophage chemotaxis and phagocytosis, enabling clearance of planktonic bacteria released from the disrupted biofilm.^221^ Another platform developed to reverse the hypoxic microenvironment of biofilms and enhance bacterial cuproptosis-like death also stimulated suppressed dendritic cells and macrophages, thereby strengthening antimicrobial activity.^222^ These findings indicate that cellular events associated with cuproptosis, or cuproptosis-like death in bacteria, can function as signals that activate and modulate innate immune cells such as macrophages and dendritic cells.

Within a chronic inflammatory microenvironment, cuproptosis and ferroptosis are unlikely to act independently. Instead, they may interact and cooperate in promoting pathological changes. Their shared links to mitochondrial metabolism and oxidative stress provide a mechanistic basis for this crosstalk.^20^

Chronic inflammation is characterized by oxidative stress, immune dysregulation, and metabolic reprogramming, all of which are central regulators of ferroptosis.^213^ Inflammatory cytokines can directly modulate expression of major ferroptosis-related proteins. As observed in rheumatoid arthritis, TNF protects synovial fibroblasts from ferroptosis, demonstrating a direct connection between inflammatory signaling and the ferroptotic machinery.^216^ Conversely, ferroptotic cells release damage-associated molecular patterns and oxidized lipids that activate inflammatory pathways such as the NOD-like receptor family pyrin domain containing 3 (NLRP3) inflammasome. This creates a self-amplifying loop between cell death and inflammation.^223^

Metabolic shifts during chronic inflammation may also sensitize cells to both cuproptosis and ferroptosis. Activated immune cells undergo marked metabolic reprogramming and often shift toward glycolysis.^224^ Because cuproptosis depends on mitochondrial respiration and the TCA cycle, highly glycolytic cells may be more resistant to this pathway. Conditions that force greater reliance on mitochondrial metabolism may, in contrast, increase vulnerability to cuproptosis.^224^ This relationship is already being exploited in cancer therapy, where tumor metabolism is being manipulated to increase sensitivity to cuproptosis inducers.^224^ A similar principle may also apply to inflammatory disease, in which the metabolic state of selected immune cell populations may be targeted to induce selective cell death. In this context, nanomedicine capable of co-delivering agents that induce both cuproptosis and ferroptosis represents an emerging frontier and may offer a more effective strategy for eliminating pathogenic cells in autoimmune and inflammatory diseases.^191^

### Metabolic diseases

Compelling evidence suggests that cuproptosis may contribute to Wilson’s disease, a hereditary disorder characterized by systemic copper accumulation caused by ATP7B mutations. Although copper overload in Wilson’s disease has traditionally been attributed to radical-mediated oxidative injury, emerging findings indicate that cuproptosis-like mechanisms may also participate in its pathogenesis.^225,226^ A recent comprehensive review of Wilson’s disease-associated liver fibrosis identifies cuproptosis as a major form of hepatocyte death.^225^ The review further proposes that early oxidative stress may create conditions that favor cuproptosis initiation. Sustained cuproptosis may then exacerbate mitochondrial dysfunction and amplify hepatocellular injury.^225^ An experimental study using primary rat astrocytes showed that toxic Cu^2+^ concentrations induce features consistent with cuproptosis. These features include the loss of mitochondrial membrane potential, nucleolar stress, and p53 accumulation. These findings indicate that cuproptosis may contribute to the neurodegenerative component of Wilson’s disease.^226^ Taken together, these observations suggest that copper-catalyzed oxidative stress and cuproptosis are not mutually exclusive in Wilson’s disease. Instead, they may represent interacting mechanisms of copper toxicity. Their relative dominance depends on copper bioavailability, subcellular copper localization, mitochondrial activity, lipoylated protein abundance, and antioxidant capacity.^225,226^

Other metabolic diseases, including obesity, type 2 diabetes, and metabolic dysfunction-associated steatotic liver disease (MASLD, previously termed nonalcoholic fatty liver disease), are characterized by systemic metabolic dysregulation, chronic low-grade inflammation, and insulin resistance.^227,228^ The liver is a major target in MASLD. This condition affects a substantial portion of adults, especially those with obesity or type 2 diabetes.^229^ Ferroptosis is increasingly recognized as a relevant mechanism in this setting. In MASLD, hepatic fat accumulation creates a lipotoxic environment that promotes oxidative stress and iron overload, thereby predisposing hepatocytes to ferroptosis. This form of cell death may contribute to progression from simple steatosis to metabolic dysfunction-associated steatohepatitis, fibrosis, and cirrhosis.^46,230^ However, conflicting evidence has also been reported. Liver parenchymal cell–specific knockout of the ferroptosis-promoting enzyme ACSL4 did not protect mice from diet-induced metabolic syndrome, MASLD, or associated hepatic injury, despite effectively reducing lipid peroxidation.^231^ These findings challenge the proposed central role of hepatocyte ferroptosis in disease progression and highlight substantial discrepancies among studies, potentially arising from differences in experimental models, dietary regimens, or disease stage.^231^

The pathogenesis of type 2 diabetes and its complications is closely linked to ferroptosis. Hyperglycemia, insulin resistance, and dyslipidemia increase oxidative stress and lipid accumulation in multiple tissues, thereby predisposing them to ferroptotic cell death.^232^ Ferroptosis is an important contributor to cardiomyocyte loss and cardiac dysfunction. Expression of the G protein-coupled receptor containing leucine-rich repeats 6 (LGR6) has been shown to be protective in this context; LGR6 alleviates ferroptosis and mitochondrial dysfunction by regulating the signal transducer and activator of transcription 3 (STAT3)/peroxisome proliferator-activated receptor gamma coactivator-1a (Pgc1a) signaling axis, which controls mitochondrial biogenesis.^192^ Another kinase, mitogen-activated protein kinase kinase kinase kinase 4 (MAP4K4), is upregulated in diabetic cardiomyopathy and exacerbates cardiac microvascular injury by promoting the S-nitrosylation of dynamin-related protein 1. This modification causes mitochondrial dysfunction and stimulates endothelial ferroptosis, thereby contributing to diabetic microvascular complications.^233^ Direct evidence linking cuproptosis to type 2 diabetes remains limited. However, cuproptosis has been associated with diabetic complications, including diabetic retinopathy^234^, cardiomyopathy^235^, and foot ulcers.^236^

The central energy sensor adenosine monophosphate-activated protein kinase (AMPK) lies at the interface between nutrient sensing and cell survival pathways, including ferroptosis. The AMPK integrates signals related to energy stores and nutrient availability to coordinate metabolic function. Its role in the pathophysiology of obesity, type 2 diabetes, and MASLD is well-established, and its ability to modulate ferroptosis suggests it may represent an important link between metabolic dysregulation and cell death in these conditions.^237^

### Cancer

Cuproptosis and ferroptosis represent unique vulnerabilities in cancer cells that may be exploited to overcome the limitations of conventional therapy, such as apoptosis resistance and drug resistance.^12,238^ Cancer cells, particularly those that have become therapy-resistant or have undergone metabolic reprogramming, often display marked sensitivity to these forms of cell death. Effective therapeutic use of these pathways remains challenging, however, because they can also exert context-dependent effects on the tumor microenvironment and immune responses.^176^

An important area of investigation concerns resistance to targeted therapy. Resistance to KRAS inhibitors, for example, can be overcome by targeting aldehyde dehydrogenase 1 family member A1 (ALDH1A1), an enzyme that protects cancer cells from ferroptosis by detoxifying aldehydes and activating the cyclic adenosine monophosphate responsive element binding protein 1 (CREB1)/GPX4 pathway.^239^ Similarly, resistance to tyrosine kinase inhibitors such as sorafenib in hepatocellular carcinoma may be circumvented by targeting the unconventional prefoldin RPB5 interactor/p53/SCD1 axis. Unconventional prefoldin RPB5 interactor promotes degradation of p53, which in turn relieves transcriptional repression of SCD1, an enzyme that produces monounsaturated fatty acids and protects cells from ferroptosis. Combining a tyrosine kinase inhibitor with an SCD1 inhibitor has shown promising anti-tumor effects in p53 wild-type hepatocellular carcinoma models.^240^

Ferroptosis induction can also be combined with other treatment modalities. In hepatocellular carcinoma, suppressor of cytokine signaling 2 (SOCS2) promotes radiosensitivity by enhancing ubiquitination and degradation of the cystine transporter SLC7A11, thereby triggering ferroptosis.^241^ Combined targeting of cyclin-dependent kinase 4 and 6 and bromodomain-containing protein 4 induces senescence in cancer cells. These senescent cells increase GPX4 expression to protect against ROS. This adaptive response creates a secondary vulnerability, rendering the cells highly sensitive to subsequent GPX4 inhibition, which triggers extensive ferroptotic cell death and tumor regression.^242^

The role of ferroptosis in cancer is not uniformly tumor suppressive. Ferroptotic cancer cells can release oxidized phospholipids, hyaluronan fragments, and other signals that create an inflammatory and pro-metastatic tumor microenvironment. In hepatocellular carcinoma, this ferroptosis-associated inflammation activates a macrophage/interleukin-1β/neutrophil axis that promotes aggressive tumor growth, lung metastasis, and resistance to sorafenib.^243^ Targeting this inflammatory axis can suppress metastasis and improve the efficacy of sorafenib.^243^

Several types of cancer also display specific vulnerabilities linked to cuproptosis. Acute myeloid leukemia, especially leukemic stem cells, depends on de novo heme biosynthesis. Inhibition of this pathway causes collapse of mitochondrial complex IV and dysregulation of the copper-chaperone system, thereby triggering cuproptosis.^244^ MYC-driven group-3 medulloblastomas, an aggressive type of pediatric brain cancer, show upregulation of DLAT, a lipoylated protein and functions centrally in cuproptosis.^245^ This upregulation sensitizes cancer cells to cell death induced by the copper ionophore elesclomol, which has shown efficacy in animal models.^245^

The tumor suppressor p53 has also been linked to cuproptosis regulation. Wild-type p53 induces the expression of a circular RNA, circFRMD4A, which inactivates the glycolytic enzyme pyruvate kinase M2.^246^ This metabolic shift redirects glycolytic flux towards the TCA cycle, increases reliance on mitochondrial metabolism, and enhances the sensitivity of cancer cells to elesclomol-induced cuproptosis.^246^ These findings suggest a potential combination strategy involving p53 agonists and cuproptosis inducers in cancers with wild-type p53. Strategies are also being developed to reprogram glycolysis-dependent tumors toward greater mitochondrial reliance, thereby increasing their susceptibility to cuproptosis. A peptide-based nanoparticle has been designed to deliver copper while simultaneously reprogramming tumor metabolism; this design substantially enhances the efficacy of cuproptosis-based therapy.^247^

### Oral and craniofacial diseases

Emerging evidence indicates that cuproptosis and ferroptosis have important pathological relevance in oral and craniofacial diseases. In periodontitis, copper chelation with tetrathiomolybdate markedly attenuates disease severity by inhibiting cuproptosis in macrophages, an effect linked to restoration of autophagic flux and lysosomal function.^110^ This intervention not only reduced cuproptosis markers but also decreased cathepsin B levels, further indicating crosstalk among cuproptosis, mitophagy, and lysosomal pathways.^110^

Recent evidence has identified copper overload and cuproptosis activation in human pulpitis tissues. This finding indicates a copper-dependent metabolic vulnerability in endodontic disease.^248^ In pulpitis, bacterial lipoteichoic acid or lipopolysaccharide triggers copper deposition and cuproptosis by directly impeding the pentose phosphate pathway. This depletes NADPH and GSH and reduces the viability of preodontoblast-like cells.^249^ A more detailed mechanistic study in chronic apical periodontitis showed that excess copper directly aggravates osteoclastogenesis and bone resorption.^250^ Copper overload inhibits glycogen synthesis via epigenetic repression of glycogen synthase 1 by histone 3 lysine 27 trimethylation (H3K27me3).^250^ This metabolic blockade reduces the availability of glucose-6-phosphate for the pentose phosphate pathway, lowers NADPH production and GSH synthesis, thereby compromising macrophage survival and exacerbating both cuproptosis and bone resorption.^250^ Collectively, these findings establish a mechanistic link among copper homeostasis, epigenetic regulation, glycogen metabolism, and oral and craniofacial diseases.

In periodontitis, proteomic analyses of patient samples have identified a ferroptosis-related protein signature (including alpha-synuclein, FTH1, and heat shock protein beta-1) that is associated with rapid disease progression, thereby linking this form of cell death to clinical outcomes.^251^ The mechanisms underlying ferroptosis in these inflammatory environments are complex. In apical periodontitis, ferroptosis is not simply a consequence of inflammation but actively contributes to bone loss. Ferroptotic macrophages release TNF-α, which in turn suppresses the osteogenic capacity of bone marrow stromal cells via the Nrf2/FSP1/ROS signaling pathway.^252^ This establishes a paracrine loop that perpetuates tissue destruction.^252^ Consistent with this concept, ferroptosis of periodontal ligament stem cells, triggered by oxidative stress and iron overload, has also been implicated in periodontitis pathogenesis.^253^

Targeting ferroptosis in these conditions has substantial therapeutic potential. The natural flavonoid neobavaisoflavone directly binds kelch-like ECH-associated protein 1, stabilizes Nrf2, and increases expression of ferroportin, thereby reducing intracellular iron, suppressing ferroptosis, and mitigating alveolar bone loss in a murine model.^254^ Multifunctional nanomaterials such as molybdenum disulfide nanoflowers functionalized with L-cysteine and galangin have also been developed for periodontitis therapy.^253^ These materials scavenge ROS, reduce iron overload, and modulate lipid peroxidation through the AMPK/Nrf2/SLC7A11 pathway, thereby providing a tailored ferroptosis-regulating strategy.^253^ In pulpitis, single-cell RNA sequencing has revealed cellular heterogeneity and confirmed ferroptosis occurrence in inflamed dental pulp cells.^255^ Thymosin α1 was found to reverse ferroptosis-related gene expression and reduce inflammation in rat models.^255^

Ferroptosis also has a major role in radiation-induced oral and craniofacial disease by promoting cell death and tissue dysfunction in several cellular compartments. In irradiated endothelial cells, lysosomal dysregulation impairs iron homeostasis and induces ferroptosis, thereby compromising angiogenic capacity and delaying tissue repair.^256^ In the oral mucosa, ionizing radiation induces ferroptosis in basal epithelial cells through activation of the spermidine/spermine N1-acetyltransferase 1 (SAT1)/ACSL4 axis, resulting in epithelial thinning and mucositis.^257^ These findings identify ferroptosis as an important mechanism of radiation injury in both endothelial and epithelial tissues of the oral and craniofacial region.

### Infectious diseases and other emerging frontiers

Recent advances have clarified the distinct and complex roles of cuproptosis and ferroptosis in infectious diseases and other emerging pathological conditions. *Mycobacterium tuberculosis* induces cuproptosis in macrophages via the LncRNA-Gm5532/miR-7232-5p/FDX1 axis, thereby promoting intracellular survival and immune evasion.^258^ Conversely, induction of cuproptosis may also be therapeutically useful. Integration of cuproptosis with cell wall digestion, for example, enhances antifungal efficacy.^259^ In sepsis, the role of cuproptosis appears to be cell type-specific. In macrophages, cuproptosis appears to be suppressed, which promotes a pro-inflammatory state.^260^ In T cells, downregulation of cuproptosis-related genes appears to mark immune dysregulation.^261^ In coronavirus disease 2019 (COVID-19), it has been proposed that infection may trigger cuproptosis in immune cells through glutamine depletion, with subsequent GSH depletion, copper overload, and mitochondrial damage.^262^

Ferroptosis has been identified as an important mechanism in COVID-19, where it contributes to acute respiratory distress syndrome and multi-organ failure by inducing the death of lung epithelial cells.^263,264^ Ferroptosis plays a dual role in infectious diseases. On one hand, it may function as a host defense mechanism by eliminating pathogen-infected cells. On the other hand, certain pathogens may exploit ferroptosis to promote tissue damage and evade immune responses.^265,266^ In fungal infection, for example, *Cryptococcus neoformans* induces ferroptosis in a *Caenorhabditis elegans* host model; the natural compound isobavachalcone protects the host by inhibiting this process, suggesting that ferroptosis modulation may represent a novel anti-infective strategy.^267^ A similar pathogenic interaction is observed in leishmaniasis, where the parasite manipulates host redox systems and creates a dynamic tension between ferroptosis and the protective Nrf2 antioxidant pathway.^268^ In viral hepatitis, both hepatitis B virus and hepatitis C virus modulate host ferroptosis pathways to establish persistent infection and promote hepatocellular carcinoma, making ferroptosis a potential antiviral target.^269,270^ Sepsis further illustrates the detrimental effects of ferroptosis. During the systemic inflammatory response, ferroptotic cell death occurs in multiple organs and contributes to tissue injury, organ dysfunction, and worsening septic shock.^271–273^

The involvement of cuproptosis and ferroptosis is also being investigated in a growing range of noninfectious disorders. In liver fibrosis, diallyl trisulfide, a garlic-derived compound, triggers cuproptosis in hepatic stellate cells through a mechanism involving Ras-related protein Rab-18 phase separation. This response inhibits lipophagy and promotes dihydrolipoamide dehydrogenase succinylation, thereby exerting an antifibrotic effect.^274^ In pulmonary fibrosis, however, cuproptosis appears to contribute to pathology by enabling M2 macrophages to resist cell death through cyclin dependent kinase inhibitor 2A-mediated mechanisms, thereby sustaining the fibrotic environment.^275^

In chronic obstructive pulmonary disease, ferroptosis-related pathways in both airway and alveolar epithelial cells contribute to airway remodeling and emphysema development.^276,277^ Ferroptosis has a dual role in pulmonary fibrosis. It promotes early epithelial cell death that initiates injury. Ferroptosis also facilitates fibroblast activation through iron accumulation, thereby advancing fibrosis.^278,279^ A similar profibrotic pattern is observed in the liver, where ferroptosis in hepatocytes and macrophages promotes fibrosis and cirrhosis.^280,281^

Together, these findings indicate that cuproptosis and ferroptosis may function either as harmful drivers of disease or as beneficial therapeutic targets, depending on the disease setting, the affected cell type, and the broader pathological context. Table 1 summarizes the major conditions associated with cuproptosis and ferroptosis.

**Table 1:** Cuproptosis and ferroptosis in diseases

Diseases	Cuproptosis	C-Ref	Ferroptosis	F-Ref
Cardiovascular diseases				
Atherosclerosis	Cuproptosis, mediated by the C1qbp–DLAT axis, contributes to the progression of atherosclerosis.	^332^	Endothelial ferroptosis triggered by iron overload and lipid peroxidation exacerbates vascular inflammation and plaque formation.	^333,334^
Cardiomyopathy	Cuproptosis may be involved in the pathogenesis of dilated cardiomyopathy through immune regulation.	^335^	Doxorubicin-induced cardiomyopathy and diabetic cardiomyopathy involve ferroptosis through mitochondrial iron accumulation and GPX4 inhibition.	^336,337^
Heart failure	Cuproptosis promotes cardiomyocyte death via copper ion dyshomeostasis and Sirtuin3 dysregulation, thereby exacerbating myocardial remodeling and heart failure.	^338^	NCOA4-mediated ferritinophagy triggers ferroptosis in cardiomyocytes, thereby accelerating cardiac dysfunction and adverse remodeling after injury.	^339,340^
Hypertension	In hypertension, downregulation of SIRT7 triggers excessive cuproptosis in the heart, which drives mitochondrial injury, myocardial fibrosis, and cardiac dysfunction.	^341^	Hypertension induces ferroptosis through the STING/ACSL4 axis, which is activated by mitochondrial DNA leakage.	^342^
Ischemia–reperfusion injury	Promotes cardiomyocyte cuproptosis during ischemia–reperfusion injury through copper-mediated oxidative stress and mitochondrial dysfunction.	^107,343^	Ferroptosis occurs predominantly during the reperfusion phase. This process is mediated by ACSL4 elevation, GPX4 degradation, and iron-catalyzed lipid peroxidation.	^186,187,344,345^
Myocardial infarction	In myocardial infarction, cuproptosis mechanistically involves copper-dependent mitochondrial dysfunction that crosstalks with mitophagy, mediated by copper-regulatory genes and immune-related hub genes.	^346,347^	Ferroptosis is a predominant form of cardiomyocyte death during ischemia–reperfusion injury and significantly increases infarct size, thereby compromising cardiac function.	^348,349^
Stroke	In intracerebral hemorrhage, copper overload triggers neuronal cuproptosis through FDX1 upregulation, loss of lipoylated proteins, and mitochondrial dysfunction, while copper chelation or FDX1 knockdown inhibits this pathway to alleviate secondary brain injury.	^350^	Cerebral ischemia–reperfusion injury triggers ferroptosis in neurons and glial cells through iron-dependent lipid peroxidation.	^351,352^
Neurodegenerative diseases				
Alzheimer’s disease	Cuproptosis, mediated through the PTBP1/SLC31A1 regulatory axis, contributes to Alzheimer’s disease pathogenesis by promoting neuronal death and amplifying oxidative stress.	^353^	Iron accumulation in the brain triggers ferroptosis of neurons and glial cells, accelerating amyloid-beta deposition and tau phosphorylation, thereby exacerbating cognitive decline and neurodegeneration.	^202,354,355^
Amyotrophic lateral sclerosis	Cuproptosis is implicated in amyotrophic lateral sclerosis pathogenesis and is associated with distinct pathological clusters characterized by unique immune and biological profiles.	^356^	Motor neuron degeneration in amyotrophic lateral sclerosis involves ferroptotic cell death mechanisms linked to iron accumulation, glutathione depletion, and lipid peroxidation.	^357,358^
Huntington’s disease	Unknown yet		Mutant huntingtin protein induces ferroptosis through impaired iron homeostasis and oxidative stress in striatal neurons.	^359,360^
Parkinson’s disease	Cuproptosis contributes to Parkinson’s disease pathogenesis by promoting death of dopaminergic neurons and is mechanistically linked to α-synuclein pathology.	^361^	Dopaminergic neuron death involves ferroptosis, characterized by iron deposition in the substantia nigra and mitochondrial dysfunction.	^362,363^
Autoimmune and inflammatory diseases				
Inflammatory bowel disease	LOXL2 promotes ulcerative colitis progression by modulating cuproptosis through the MAPK signaling pathway.	^364^	Intestinal epithelial cell ferroptosis exacerbates mucosal barrier dysfunction and inflammatory responses in colitis.	^365,366^
Multiple sclerosis	Unknown yet		In multiple sclerosis, neuronal ferroptosis is initiated by the degradation of GPX4, linking inflammatory signaling to oxidative damage.	^367,368^
Osteoarthritis	In osteoarthritis, elevated oxygen levels induce copper overload by upregulating the copper importer SLC31A1 and downregulating the copper exporter ATP7B, which triggers DLAT oligomerization-dependent cuproptosis and accelerates cartilage degeneration.	^369^	Mechanical stress and other inducers trigger GPX4-regulated ferroptosis in chondrocytes, leading to extracellular matrix degradation and osteoarthritis progression.	^370,371^
Psoriasis	Cuproptosis has a complex role in psoriasis, exerting therapeutic effects by inhibiting keratinocyte proliferation through induced copper overload, but also aggravating inflammation through the SLC31A1/KDM5B/FTH1 axis that promotes ferroptosis.	^119,372^	Keratinocyte ferroptosis modulates skin inflammation through lipid peroxidation pathways.	^373,374^
Rheumatoid arthritis	Cuproptosis is increasingly implicated in rheumatoid arthritis, and suppression of this pathway in chondrocytes through mechanisms such as the METTL3/miR-221/222-3p/ATP7A axis reduces joint damage and inflammation.	^375,376^	Ferroptosis of synovial fibroblasts and macrophages drives joint inflammation and bone destruction in rheumatoid arthritis.	^218,377^
Systemic lupus erythematosus	Cuproptosis-related genes are closely associated with immune cell infiltration in systemic lupus erythematosus.	^378^	Neutrophil ferroptosis serves as an essential driver of neutropenia and tissue damage in lupus pathogenesis.	^163,379^
Metabolic diseases				
Diabetic complications	In diabetic cardiomyopathy, hyperglycemia-induced cardiac copper overload triggers cuproptosis, disrupting the mitochondrial lipoylation process.	^235^	Ferroptosis, triggered by impaired mitochondria-associated endoplasmic reticulum membrane integrity, drives renal tubular injury in diabetic nephropathy.	^380^
Insulin resistance	Unknown yet		Skeletal muscle and hepatic ferroptosis impair insulin signaling and glucose uptake, establishing a mechanistic link between iron-dependent cell death and the development of insulin resistance.	^381–383^
Iron overload disorders	Unknown yet		Iron chelators chelate excess iron to treat iron overload disorders and prevent ferroptosis.	^384,385^
Metabolic syndrome	Unknown yet		Tissue-specific ferroptosis contributes to the multi-organ manifestations of metabolic syndrome, including hepatic, cardiovascular, and renal complications.	^386,387^
NAFLD/MASLD	Cuproptosis is implicated in the pathogenesis of NAFLD through the dysregulation of key cuproptosis-related genes such as DLAT.	^388^	Some studies have supported that hepatocyte ferroptosis promotes steatosis-to-steatohepatitis progression via lipid peroxidation and iron overload. However, contradictory evidence exists, as liver parenchymal cell–specific knockout of the ferroptosis-promoting enzyme ACSL4 failed to protect mice from diet-induced MASLD.	^231,321,389,390^
Obesity	In obesity, a high-fat diet induces copper overload and upregulates the cuproptosis-executing genes FDX1 and DLAT, thereby triggering cuproptosis in the liver and kidney.	^391^	Ferroptosis regulates metabolic inflammation and insulin resistance in obese states.	^381,392^
Type 2 diabetes	Unknown yet		Ferroptosis mediates pancreatic β-cell damage and dysfunction in type 2 diabetes.	^393^
Wilson’s disease	In Wilson’s disease, copper accumulation promotes cuproptosis through disruption of redox homeostasis and induction of nucleolar stress, thereby worsening hepatic and neurological pathology.	^225,226^	Unknown yet	
Cancer				
Bladder cancer	Cuproptosis functions both as an important regulatory mechanism in bladder cancer through LIPT1-mediated metabolic reprogramming and endoplasmic reticulum stress, and as a therapeutic target via nanomedicine-enabled copper delivery.	^394,395^	Ferroptosis, triggered by a bacterial metabolite, plays a decisive immunomodulatory role in bladder cancer by removing pro-tumorigenic neutrophils, which underlies a crucial mechanism for the better clinical outcome in females.	^396^
Breast cancer	Cuproptosis represents an emerging therapeutic strategy in breast cancer through targeting copper homeostasis, modulating the TME to enhance immunogenic cell death, and interacting with critical signaling pathways such as NF-κB to overcome therapy resistance.	^397–399^	Elevated expression of FADS1/2 in TNBC cells promotes the biosynthesis of PUFAs, thereby enhancing cellular susceptibility to ferroptosis. In contrast, estrogen receptor-positive breast cancers are generally relatively insensitive to ferroptosis inducers. However, endocrine therapy can restore sensitivity of estrogen receptor-positive cancer cells to ferroptosis inducers.	^400–402^
Cervical cancer	Cuproptosis is a druggable vulnerability that can be selectively induced in cervical cancer through disruption of copper homeostasis.	^403^	Inhibition of ferroptosis promotes progression of cervical cancer.	^404,405^
Colorectal cancer	Integration of cuproptosis and hypoxia profiles stratifies colorectal cancer into distinct molecular subtypes with heterogeneous metabolic landscapes, immune microenvironments, and drug sensitivities, thereby informing personalized therapeutic strategies.	^406^	Ferroptosis induction overcomes chemotherapy resistance in colorectal cancer through lipid peroxidation modulation.	^407,408^
Esophageal cancer	In esophageal cancer, radiotherapy induces cuproptosis through mitochondrial copper accumulation. Resistance arises from BACH1 downregulation-driven metallothionein expression, which sequesters copper. This vulnerability may be targeted with copper ionophores to restore radiosensitivity.	^309^	In esophageal adenocarcinoma, inhibition of ferroptosis contributes to radioresistance.	^409^
Gastric cancer	Cuproptosis signaling contributes to gastric cancer heterogeneity through distinct molecular subtypes and is associated with differences in clinical outcomes and molecular landscapes.	^410^	Gastric cancer cells evade ferroptosis by upregulating defense mechanisms such as GPX4, thereby promoting chemoresistance and metastatic progression.	^411,412^
Glioma	Cuproptosis is being explored in glioma therapy through nanoplatform-enabled copper delivery to overcome therapeutic barriers, synergize with apoptosis, remodel the TME, and activate antitumor immunity, thereby suppressing tumor progression and postoperative recurrence.	^413–415^	CDKN2A deletion in glioblastoma remodels lipid metabolism by redistributing PUFAs, elevating lipid peroxidation, and selectively sensitizing tumors to ferroptosis, revealing a therapeutic vulnerability.	^416^
HCC	Cuproptosis represents an important therapeutic mechanism in HCC, the activation of which is context-dependent. This pathway may be enhanced through metabolic reprogramming, dual-ion interference, or targeted sensitization strategies to overcome therapeutic resistance and remodel the immunosuppressive TME.	^417–419^	Ferroptosis suppression by the zone 3‑specific genes Gstm2 and Gstm3 contributes to HCC initiation.	^420^
Leukemia	Cuproptosis is induced by inhibition of heme biosynthesis in AML cells, particularly leukemic stem cells, through the collapse of mitochondrial complex IV and dysregulation of the copper chaperone system.	^244^	Ferroptosis susceptibility in leukemia is not uniform but is instead context-dependent, presenting both a mechanism of resistance and a therapeutic opportunity.	^421,422^
Lung cancer	Cuproptosis is suppressed by oncogenic circRNAs such as circKIAA1797 to promote lung cancer progression. In contrast, targeted metabolic interventions that enhance cuproptosis represent promising strategies to increase immunotherapy sensitivity and inhibit tumor growth.	^423–425^	FSP1-mediated ferroptosis suppression is essential for lung cancer tumorigenesis, rendering FSP1 inhibition a promising therapeutic strategy to trigger ferroptotic cell death.	^426^
Lymphoma	In lymphoma, cuproptosis has an important role both as a prognostic indicator, through cuproptosis-related gene signatures, and as a therapeutic target, with its induction by copper ionophores or nano-sonosensitizers enhancing antitumor immunity.	^427,428^	On the one hand, certain metabolic products can protect lymphoma cells from ferroptosis, leading to a more aggressive phenotype and treatment resistance. On the other hand, drug-induced ferroptosis effectively eradicates lymphoma cells.	^93,429^
Melanoma	Cuproptosis represents an emerging therapeutic strategy in melanoma that not only directly suppresses tumor growth but also enhances immunotherapy through activation of the cGAS-STING pathway and induction of immunogenic cell death.	^430,431^	In the hypoxic lymph node metastatic niche, melanoma shifts its ferroptosis suppression dependency from GPX4 to FSP1, rendering it specifically vulnerable to FSP1 inhibition.	^432^
Oral squamous cell carcinoma	Induction of cuproptosis through targeted regulation of metabolic and genetic pathways suppresses tumor growth and improves immunotherapy efficacy in oral squamous cell carcinoma.	^433,434^	Inhibition of ferroptosis promotes oral squamous cell carcinoma progression.	^435,436^
Osteosarcoma	Although copper-based nanomaterials have been used to induce immunogenic cell death and remodel the TME in osteosarcoma therapy, whether their therapeutic effects are mediated by oxidative stress and mitochondrial dysfunction or by cuproptosis remains context-dependent.	^437,438^	Ferroptosis serves as a critical tumor-suppressive mechanism in osteosarcoma, where its induction effectively inhibits cancer cell proliferation and progression.	^439,440^
Ovarian cancer	Cuproptosis-related genes, particularly *GJB2*, play a dual role in ovarian cancer by regulating copper homeostasis and tumor progression, and by modulating immune evasion, with potential value as prognostic biomarkers and therapeutic targets.	^441^	Ferroptosis susceptibility, driven by PUFA-lipid accumulation via ACSL4, promotes ovarian cancer metastasis by enhancing membrane fluidity and invasiveness.	^66^
Pancreatic cancer	In pancreatic cancer, cuproptosis serves as both a prognostic indicator through cuproptosis-related lncRNAs associated with immune infiltration and drug sensitivity, and a theranostic target, with nanomaterial-mediated copper ion delivery enhancing the efficacy of immunotherapy.	^442,443^	In pancreatic cancer, cystine import via system x_c_^-^ fuels glutathione synthesis to suppress ferroptosis, while targeting this pathway induces tumor-selective ferroptosis and inhibits cancer growth.	^444^
Prostate cancer	Cuproptosis represents a promising therapeutic strategy in prostate cancer, particularly in the setting of enzalutamide resistance. This is because synergistic induction of cuproptosis by enzalutamide and copper ionophores can overcome resistance.	^100,445^	ALKBH5-mediated regulation of the CHRM3/ZNF281 axis sensitizes prostate cancer cells to ferroptosis, revealing a novel tumor-suppressive mechanism and therapeutic vulnerability in castration-resistant prostate cancer.	^446^
Oral and craniofacial diseases				
Dental pulp and periapical diseases	In both pulpitis and chronic apical periodontitis, bacterial component-induced copper overload triggers cuproptosis by impairing the pentose phosphate pathway, thereby depleting NADPH and glutathione and exacerbating inflammatory tissue damage.	^249,250^	Ferroptosis contributes to pulpitis and periapical lesion formation through iron-dependent oxidative stress in dental pulp cells and macrophages, influencing endodontic treatment outcomes.	^252,255^
Periodontitis	Cuproptosis contributes to periodontitis by inducing lysosomal damage and subsequent blockade of autophagic flux in macrophages, thereby exacerbating periodontal inflammation and tissue destruction.	^110^	Gingival fibroblasts and osteoclasts ferroptosis exacerbate alveolar bone loss and connective tissue destruction.	^251,253,254^
Radiation-induced oral and craniofacial diseases	Unknown yet		Epithelial ferroptosis triggers radiation-induced oral mucositis, while endothelial ferroptosis promotes radiation-induced mandibular regeneration disorder.	^256,257,447^
Infectious diseases				
COVID-19	SARS-CoV-2 infection may trigger cuproptosis in immune cells by reducing glutamine levels. This results in glutathione depletion, copper overload, and mitochondrial damage.	^262^	SARS-CoV-2 infection induces ferroptosis in lung epithelial cells, contributing to acute respiratory distress syndrome and multi-organ failure.	^263,264^
Fungal infection	Cuproptosis enhances antifungal efficacy through integration with cell wall digestion, thereby increasing fungal killing.	^259^	Fungal pathogens induce ferroptosis in immune cells, modulating host defense mechanisms.	^448^
Leishmaniasis	Unknown yet		Ferroptosis and Nrf2 signaling play a redox tug-of-war in leishmaniasis pathogenesis.	^268^
Sepsis	In sepsis, cuproptosis appears to be suppressed in macrophages, which promotes a pro-inflammatory state. In T cells, downregulation of cuproptosis-related genes appears to mark immune dysregulation. These contrasting observations suggest that cuproptosis involvement is cell type-specific.	^260,261^	Systemic inflammatory response triggers ferroptosis in multiple organ systems, exacerbating septic shock and tissue damage.	^271–273^
Tuberculosis	*Mycobacterium tuberculosis* induces cuproptosis through the LncRNA-Gm5532/miR-7232-5p/FDX1 axis, thereby enhancing intracellular survival and immune evasion in macrophages.	^258^	Ferroptosis serves as a pathogen-exploited cell death mechanism in tuberculosis.	^265,266^
Viral hepatitis	Unknown yet		Hepatitis B and C viruses modulate host ferroptosis pathways to promote viral persistence and HCC development, with ferroptosis representing a potential antiviral target.	^269,270^
Other diseases				
COPD	In COPD, cigarette smoke-induced copper accumulation and downregulation of the cuproptosis-related gene glutaminase in alveolar macrophages trigger cuproptosis, thereby impairing macrophage function and contributing to disease pathogenesis.	^449,450^	The ferroptosis-driven pathways in airway epithelial cells (causing airway remodeling) and alveolar epithelial cells (causing emphysema) collectively contribute to COPD.	^276,277^
Epilepsy	In temporal lobe epilepsy, copper dyshomeostasis upregulates the cuproptosis-related genes LIPT1 and FDX1, which disrupt the TCA cycle and pyruvate metabolism to induce neuronal cuproptosis.	^451^	Ferroptosis is a crucial process in temporal lobe epilepsy, and its inhibition shows therapeutic potential.	^452^
Glaucoma	Unknown yet		Lipid peroxidation is the central detrimental process in glaucoma, while the role of iron-dependent execution phase of ferroptosis may depend on the specific pathological context.	^453,454^
Liver fibrosis	In liver fibrosis, cuproptosis is triggered in hepatic stellate cells by DATs through RAB18 phase separation. This process inhibits lipophagy and promotes succinylation of dihydrolipoamide dehydrogenase to exert therapeutic effects.	^274^	Hepatocyte and macrophage ferroptosis promote liver fibrosis and cirrhosis.	^280,281^
Nanoplastic toxicity	Polystyrene nanoplastics trigger macrophage cuproptosis by stabilizing the copper importer SLC31A1, which in turn activates alveolar epithelial pyroptosis and extracellular matrix degradation, ultimately leading to emphysema.	^455^	Ferroptosis serves as a pivotal mechanism in nanoplastic-induced pulmonary toxicity	^456,457^
Pulmonary fibrosis	Cuproptosis contributes to pulmonary fibrosis by enabling M2 macrophages to resist cell death through CDKN2A-mediated mechanisms.	^275^	Ferroptosis contributes to pulmonary fibrosis by promoting epithelial cell death to initiate injury and facilitating fibroblast activation through iron accumulation.	^278,279^

*ALKBH5* alkB homolog 5 RNA demethylase, *AML* acute myeloid leukemia, *BACH1* BTB domain and CNC homolog 1, *C1qbp* complement C1q binding protein, *CDKN2A* cyclin dependent kinase inhibitor 2A, *cGAS* cyclic guanosine monophosphate-adenosine monophosphate synthase, *CHRM3* cholinergic receptor muscarinic 3, *COPD* chronic obstructive pulmonary disease, *DATs* diallyl trisulfides, *FADS1* fatty acid desaturase 1, *FADS2* fatty acid desaturase 2, *GJB2* gap junction protein beta 2, *Gstm2* glutathione S-transferase mu 2, *Gstm3* glutathione S-transferase mu 3, *HCC* hepatocellular carcinoma, *KDM5B* lysine demethylase 5B, *lncRNAs* long non-coding RNAs, *LOXL2* lysyl oxidase like 2, *MAPK* mitogen-activated protein kinase, *METTL3* methyltransferase 3 N6-adenosine-methyltransferase complex catalytic subunit, *NAFLD* nonalcoholic fatty liver disease, *PTBP1* polypyrimidine tract binding protein 1, *PUFAs* polyunsaturated fatty acids, *RAB18* Ras-related protein Rab-18, *SARS-CoV-2* severe acute respiratory syndrome coronavirus 2, *SIRT7* sirtuin 7, *TME* tumor microenvironment, *TNBC* triple-negative breast cancer, *ZNF281* zinc finger protein 281

## Therapeutic strategies and frontiers in clinical translation

The discovery and mechanistic characterization of cuproptosis and ferroptosis have created new opportunities for therapeutic intervention in a broad range of diseases. Pharmacologic induction or inhibition of these pathways offers a versatile means of modulating cell fate. Current strategies include small-molecule modulators, nanotechnology-based delivery systems, and combination approaches, with an increasing number of candidates undergoing preclinical and early clinical evaluation.

### Small-molecule modulators and tool compounds

Progress in this field has depended heavily on the identification of small molecules that selectively modulate cuproptosis and ferroptosis. These compounds have served both as indispensable mechanistic tools and as starting points for therapeutic development.

A principal strategy for inducing cuproptosis is to increase intracellular copper levels by using copper ionophores. Preclinical studies have demonstrated that several copper ionophores can trigger cuproptosis. Elesclomol and disulfiram represent the most thoroughly investigated agents within this category. Elesclomol functions primarily by selectively delivering copper to the mitochondria. It forms a 1:1 complex with extracellular Cu^2+^ and rapidly transports it into mitochondria, where Cu^2+^ is reduced to Cu^+^ by FDX1.^282,283^ This process generates sustained mitochondrial oxidative stress through redox cycling. Cuproptosis occurs when copper directly binds to lipoylated components of the TCA cycle, causing protein aggregation and proteotoxic stress.^9,282^ The cytotoxicity of elesclomol is particularly pronounced in cells with high mitochondrial respiration and low glycolytic activity, such as cancer stem cells and drug-resistant tumor cells, whereas normal cells with higher antioxidant capacity appear relatively less affected.^282,284^ Identification of cuproptosis has therefore provided a clearer mechanistic explanation for the action of elesclomol, especially in cells with high mitochondrial metabolic activity.^9^

Disulfiram is a US Food and Drug Administration (US FDA)-approved aldehyde dehydrogenase inhibitor.^285^ It exerts multifaceted antitumor effects through complex formation with copper.^286^ The disulfiram/Cu complex suppresses nuclear factor kappa-light-chain-enhancer of activated B cells (NF-κB) signaling, increases ROS production, and ultimately triggers programmed cell death.^287^

Clinical development of these cuproptosis-inducing agents has produced mixed results. In a phase II trial in stage IV melanoma, combining elesclomol with paclitaxel doubled median progression-free survival (56 vs 112 days) and improved median overall survival (11.9 vs 7.8 months) compared with paclitaxel alone.^288^ However, these outcomes were not reproduced in a subsequent large phase III randomized controlled trial, which failed to meet its primary endpoint. A retrospective analysis from that trial suggested serum lactate dehydrogenase as a potential predictive marker for treatment response.^289^ For disulfiram, a phase IIb trial in non-small cell lung cancer showed that adding disulfiram to cisplatin–vinorelbine extended median survival from 7.1 to 10 months.^290^ In contrast, a phase II/III trial involving patients with recurrent glioblastoma found no survival benefit from adding disulfiram and copper to alkylating chemotherapy. There was no improvement in either 6-month survival rate or median progression-free survival.^291^ Taken together, these studies indicate that induction of cuproptosis remains conceptually promising but has shown limited success in clinical practice. Efficacy appears restricted when these agents are used alone or within standard combination regimens, which indicates the need for new approaches that broaden and strengthen their antitumor effects.

The major inhibitors of cuproptosis are copper chelators. Tetrathiomolybdate and bathocuproinedisulfonic acid, which are membrane-impermeable and membrane-permeable chelators, respectively, can effectively block cuproptosis through copper sequestration.^41,100^

Ferroptosis inducers are commonly classified according to their mechanism of action. Class 1 inducers, such as erastin and sulfasalazine, inhibit the cystine/glutamate antiporter SLC7A11, leading to GSH depletion.^81,151^ Class 2 inducers such as ras-selective lethality protein 3 directly and covalently inhibit GPX4.^151^ Class 3 inducers such as ferroptosis inducer 56 promote degradation of GPX4 protein. Class 4 inducers, or ferroptosis-inducing compounds, act by consuming coenzyme Q10 or inhibiting its synthesis.^4^ Several approved drugs, including the multi-kinase inhibitor sorafenib, have been found to induce ferroptosis as part of their anti-cancer mechanism.^151^

The most widely used ferroptosis inhibitors are lipophilic radical-trapping antioxidants. Ferrostatin-1 and liproxstatin-1 are potent inhibitors that block the propagation of lipid peroxidation within membranes.^12^ Iron chelators such as deferoxamine and deferiprone can also inhibit ferroptosis by reducing the labile iron pool.^46^ These inhibitors are being explored for therapeutic use in disorders associated with excessive ferroptosis, including ischemia–reperfusion injury and neurodegeneration.^181,292^

Some compounds also influence the interplay between these pathways. Copper itself can modulate ferroptosis by promoting GPX4 degradation.^117^ Targeting shared regulatory nodes such as p53 or HIF-1α also represents a strategy for simultaneous modulation of both pathways.^148,175^

### Nanotechnology, targeted prodrugs, and combination strategies

Nanotechnology provides a powerful set of approaches for addressing many pharmacological limitations of small-molecule modulators of cuproptosis and ferroptosis.^293^ Nanocarriers, typically ranging from 1 to 100 nanometers in size, can encapsulate therapeutic agents, protect them from premature degradation in the bloodstream, improve solubility, and modify pharmacokinetic behavior.^293^ A major advantage of nanomedicine is its ability to exploit the enhanced permeability and retention effect, in which leaky tumor vasculature and poor lymphatic drainage promote passive accumulation of nanoparticles at tumor sites.^293^ A variety of nanomaterials, including liposomes, polymeric nanoparticles, and metal-organic frameworks, have been engineered to deliver cuproptosis- or ferroptosis-modulating agents. For example, smart liposomal nanocarriers have been designed to target ischemic brain tissue, a setting in which ferroptosis contributes to pathology. This approach illustrates the potential of disease-site-specific delivery.^294^

**Fig. 7:**
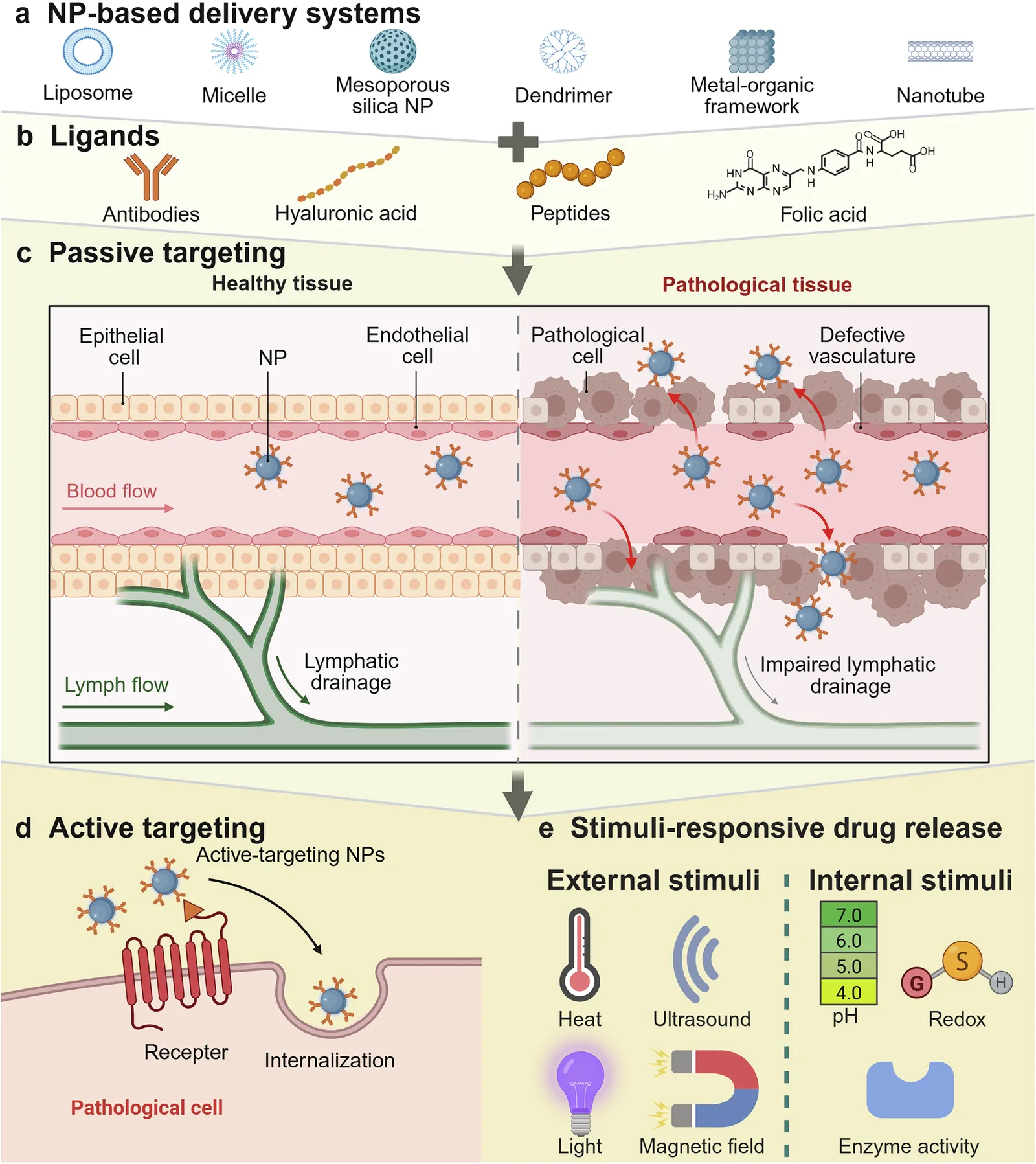
Nanotherapeutics for diseases. **a** Nanoparticle-based drug delivery systems have been developed to improve the therapeutic index of agents. These nanocarriers can encapsulate drugs, protect them from premature degradation, and reduce systemic toxicity.^459^ **b** The surfaces of nanoparticles can also be functionalized with targeting ligands such as antibodies, peptides, or aptamers. **c** By exploiting the enhanced permeability and retention effect, nanoparticles can passively accumulate in pathological tissues. **d** Active targeting of pathological cells helps to increase drug concentration at the pathological site and minimize off-target effects.^460,461^ **e** To minimize systemic toxicity and enhance tumor specificity, nanoparticles are often engineered to release their payload in response to specific triggers present in the microenvironment or applied externally. NP nanoparticle. Created in BioRender. Li, Y. (2026) https://BioRender.com/pc22unu

Nanoplatforms can also be engineered to overcome major biological barriers such as the blood-brain barrier, which prevents most therapeutics from reaching brain tumors such as glioblastoma.^295^ Strategies under active investigation include intranasal delivery through customized hydrogels and development of brain-targeting nanoparticles that engage specific receptors on blood-brain barrier endothelium to enhance central nervous system delivery.^296,297^ Nanocarriers may also be designed as stimulus-responsive systems that release their payload only in response to local triggers within the tumor microenvironment, such as low pH, elevated ROS, or specific enzymes (Fig. 7).^298^ For example, ROS-responsive nanoparticles have been developed to release a ferroptosis inhibitor prodrug selectively within the high oxidative stress environment of spinal cord injury, thereby facilitating tissue repair.^299^ Despite these advances, clinical translation of nanomedicines remains challenging. Major obstacles include difficulties in scalable manufacturing, potential immunogenicity, and limited ability to predict in vivo behavior, particularly formation of the protein corona that alters targeting efficacy and clearance.^293^

Targeted prodrugs are pharmacologically inert derivatives of active pharmaceutical agents that are engineered for selective activation by specific enzymes or membrane transporters.^300^ This design can improve bioavailability and site-specific delivery by restricting drug activation to the intended location.^300^ A dual-locked platinum(IV) prodrug has been developed to release cisplatin, a chemotherapeutic agent, and a toll-like receptor 7/8 agonist, an immunomodulator, in a sequential manner triggered by the high GSH levels and γ-glutamyltranspeptidase activity present in many tumors. This targeted release concentrates cytotoxic activity within the tumor and simultaneously induces both ferroptosis and apoptosis while stimulating an antitumor immune response.^301^ Similarly, self-assembled nanoparticles derived from a hydrogen sulfide prodrug have been developed to release the gasotransmitter hydrogen sulfide in response to high cysteine levels in glioblastoma cells, thereby inducing ferroptosis in a targeted manner.^302^ Another innovative strategy uses a cytochrome c oxidase-like nanozyme to activate the bioreductive prodrug AQ4N specifically within the hypoxic tumor microenvironment, producing a synergistic combination of starvation, ferroptosis, and chemotherapy.^303^ These approaches illustrate the potential of bioorthogonal chemistry and rational drug design for generating highly specific therapeutic agents. By confining activation of potent cell death inducers to tumors, targeted prodrugs can widen the therapeutic window and permit higher effective doses without a parallel increase in systemic toxicity.

The goal of cancer therapy is not only to eliminate tumor cells but to achieve durable disease control. Increasing evidence indicates that the mode of cancer cell death strongly influences the host antitumor immune response. Certain forms of RCD termed immunogenic cell death release a characteristic set of molecules, including damage-associated molecular patterns that function as “eat me” signals. These signals promote dendritic cell maturation and the priming of tumor-specific T cells.^304^ This process can convert an immunologically “cold” tumor, which lacks meaningful immune infiltration, into an immunologically “hot” tumor.^305^ As a result, the tumor may become more susceptible to immune checkpoint inhibitors such as anti-programmed cell death protein 1 (PD-1) or anti-programmed cell death ligand 1 (PD-L1) antibodies.^305^

Preclinical studies have shown that induction of cuproptosis and ferroptosis can enhance the efficacy of immune checkpoint inhibition. A nanoplatform designed to induce cuproptosis synergized with anti-PD-L1 therapy to improve tumor control.^306^ Likewise, several nanomedicine formulae have been developed to co-induce cuproptosis and ferroptosis together with photodynamic or sonodynamic therapy, both of which can also trigger immunogenic cell death. These multimodal strategies generate a strong synergistic effect in which initial tumor cell death primes the immune response. The checkpoint blockade subsequently amplifies that response.^307,308^

Combining cuproptosis or ferroptosis modulators with standard therapies is another promising strategy. Conventional treatments, including radiotherapy and chemotherapy, can induce cellular stress that sensitizes cancer cells to metal-dependent death. Radiotherapy increases mitochondrial copper concentrations through induction of CTR1 expression and reduction of mitochondrial GSH, thereby initiating cuproptosis.^309^ Administration of copper ionophores further increases the sensitivity of radiotherapy-resistant malignancies by enhancing cuproptosis.^309^ Ferroptosis induction with bioactive compounds also represents a viable approach for overcoming chemotherapy resistance.^310^ However, such combination strategies must account for the inherent toxicity of radiotherapy and chemotherapy to normal tissues, including their potential to contribute to second primary tumors.^311^

### Clinical translation: safety, endpoints, and trial design

Translating promising preclinical findings on cuproptosis and ferroptosis into effective clinical therapies remains a substantial challenge. A growing number of clinical studies are evaluating therapies related to these pathways. Elesclomol has been investigated in several cancer trials. Although early studies produced mixed results, improved understanding of its cuproptosis-inducing mechanism and identification of predictive biomarkers, such as high mitochondrial metabolic activity, have renewed interest in its clinical use.^282,312^ Many trials are also examining combinations of ferroptosis-modulating agents with other therapeutic modalities such as immune checkpoint blockade. For example, clinical trials are evaluating combinations of agents that modulate the kynurenine/tryptophan ratio, a metabolic pathway linked to both ferroptosis and immune regulation, with anti-PD-1 therapy.^313,314^

A central challenge is management of on-target, off-tumor toxicity. Development of tumor-targeted nanodelivery systems and tumor microenvironment-responsive smart prodrugs may improve therapeutic precision and reduce adverse effects.^315,316^ Clinical trial design for these agents also requires careful endpoint selection. In addition to conventional endpoints such as overall survival and progression-free survival, pharmacodynamic biomarkers that confirm target engagement are essential.^317^ Such biomarkers may include evidence of lipid peroxidation or DLAT aggregation in tumor biopsies. The high cost of emerging therapeutic strategies, particularly combination regimens involving immune checkpoint blockade and nanomedicine formulations, presents an additional challenge and warrants rigorous cost-effectiveness evaluation.^318,319^

### Biomarkers, stratification, and response monitoring

A major goal of precision oncology is to predict which patients are most likely to benefit from therapy. For cuproptosis- and ferroptosis-based treatment, ideal predictive biomarkers should reflect the functional state of the target pathways within the tumor. For example, tumors with high expression of lipoylated TCA cycle proteins and strong copper influx capacity may be particularly suitable for cuproptosis-inducing agents.^238^ Similarly, tumors with low expression of major ferroptosis defense proteins GPX4 or SLC7A11, or high levels of iron and polyunsaturated fatty acids, would be expected to show greater sensitivity to ferroptosis inducers.^12^

Clinical research increasingly incorporates biomarker analysis to guide therapeutic decisions. In immunotherapy, PD-L1 expression and stromal tumor-infiltrating lymphocytes are established predictive biomarkers for response to checkpoint inhibitors in several types of cancer.^320^ A comparable framework is needed for cell death-based therapies. Preclinical studies suggest that serum ferritin may serve as a predictive biomarker for ferroptosis-based therapy in metabolic dysfunction-associated steatohepatitis. The use of this biomarker offers a potentially noninvasive method for patient selection.^321^ Broader genomic signatures, including those associated with chromosomal instability or DNA damage response, have also been shown to predict resistance to conventional chemotherapy and may also be adaptable for predicting sensitivity to novel RCD inducers.^317^ Validation of such biomarkers in prospective clinical trials will be essential for clinical implementation. Such an approach will help identify patients most likely to benefit while reducing unnecessary toxicity.

Apart from the use of single biomarkers, a more complete picture of tumor biology may be acquired by integrating multiple layers of molecular information through multi-omics analysis. This approach can identify molecular subtypes that are not evident from histology alone and that may have distinct therapeutic vulnerabilities. Multi-omics profiling of human epidermal growth factor receptor 2 (HER2)-positive breast cancer has identified four distinct subtypes (classical, immunomodulatory, luminal-like, and basal/mesenchymal-like).^322^ Each of these subtypes has a different predicted sensitivity to anti-HER2 therapy, immunotherapy, or other targeted agents. This distinction provides the basis for more tailored treatment strategies.^322^ This principle is also applicable to cuproptosis and ferroptosis. A multi-omics study of hepatocellular carcinoma identified three molecular subtypes based on fatty acid degradation pathways that are closely linked to ferroptosis.^323^ These subtypes showed distinct clinical, metabolic, and immunological characteristics and, importantly, different responses to sorafenib, immunotherapy, and transarterial chemoembolization.^323^ Identification of these subtypes enables more personalized treatment allocation based on tumor metabolic profile.^323^

Single-cell RNA sequencing provides greater resolution by revealing intratumoral heterogeneity and identifying rare cell populations that may contribute to therapeutic resistance.^324^ Artificial intelligence and machine learning are increasingly important for integrating these large, complex datasets and developing predictive models for clinical decision-making.^325,326^ These technologies may advance cancer care beyond one-dimensional classification toward treatment strategies tailored to the molecular architecture of each patient’s tumor.

Understanding the molecular basis of acquired resistance will also be essential for development of effective second-line therapeutic regimens. Real-world multi-omics analyses of breast cancer treated with cyclin-dependent kinase 4/6 inhibitors have identified bifurcating evolutionary trajectories.^327^ In these patterns, tumors acquire resistance through either estrogen receptor-dependent or estrogen receptor-independent mechanisms, each requiring a different subsequent therapeutic strategy.^327^ Computational platforms such as PERCEPTION, which use single-cell transcriptomic data from individual tumors to model and predict treatment response and resistance, represent a potential future direction for adaptive personalized therapy.^328^ This approach has captured resistance development in lung cancer and has outperformed other predictive methods in clinical cohorts.^328^ Continuous monitoring of tumor molecular evolution, together with improved understanding of escape pathways, may therefore permit adaptive treatment strategies that improve long-term patient outcomes.

## Conclusions and future direction

This review provides an integrated analysis of cuproptosis and ferroptosis as mechanistically distinct yet metabolically interconnected forms of metal-dependent regulated cell death. Cuproptosis is mediated primarily by copper-induced proteotoxic stress involving lipoylated mitochondrial proteins, whereas ferroptosis results from iron-dependent lipid peroxidation. By examining these pathways together, the review identifies points of convergence involving mitochondrial metabolism, lysosomal regulation, and organelle crosstalk.

A central conclusion is that both pathways exert highly context-dependent biological effects. They may suppress tumor growth in some settings yet promote tissue injury, inflammation, or disease progression in others. These divergent outcomes are influenced by cellular metabolism, metal availability, antioxidant capacity, and microenvironmental conditions. Both pathways are governed by complex, multilayered regulatory networks involving genetic and epigenetic determinants, non-coding RNA-mediated regulation, targeted protein degradation, and post-translational modifications. Their pathophysiological relevance extends to a broad range of diseases, including cardiovascular, neurodegenerative, and metabolic disorders, as well as cancer.

Despite these advances, several fundamental questions about cuproptosis and ferroptosis remain unresolved. First, for cuproptosis, copper can catalyze Fenton-type free radical reactions, similar to iron. However, radical-mediated injury does not appear to be the primary execution mechanism of cuproptosis. Instead, cuproptosis is driven by copper-dependent aggregation of lipoylated mitochondrial proteins and loss of Fe–S cluster proteins. This distinction is particularly relevant to Wilson’s disease, a classic copper overload disorder. Although Wilson’s disease has long been associated with copper-driven oxidative stress, emerging evidence indicates that cuproptosis-like mechanisms may also occur in this setting.^225,226^ Thus, the apparent discrepancy may reflect differences in copper localization and cellular context rather than the absence of cuproptosis. Oxidative damage may dominate when excess copper remains broadly distributed or reacts with hydrogen peroxide. Cuproptosis may be initiated when bioavailable copper accumulates in mitochondria and encounters lipoylated TCA cycle proteins in metabolically active cells. In Wilson’s disease, copper-catalyzed oxidative stress and cuproptosis therefore appear to act as parallel and interacting facets of copper toxicity.^225,226^ Defining the metabolic and subcellular conditions that determine whether oxidative damage or proteotoxic stress predominates remains an important direction for future investigation. Second, the radical source in ferroptosis remains debated. Lipid peroxidation may be initiated and propagated by labile iron through Fenton chemistry, enzyme-bound iron in lipoxygenases, or other organelle-specific radical-generating systems. The intracellular source and trafficking route of the lethal iron pool also remain incompletely defined.^329^ Third, the terminal execution mechanism of ferroptosis remains unclear. Unlike apoptosis, necroptosis, and pyroptosis, ferroptosis lacks a clearly identified protein executor. How lipid peroxidation ultimately causes plasma membrane rupture also remains unresolved.^330^ Fourth, direct evidence that cuproptosis and ferroptosis occur in vivo in human disease remains limited. Copper or iron accumulation alone does not prove cuproptosis or ferroptosis. Reliable and specific biomarkers are still needed to distinguish these metal-dependent cell death pathways from other forms of oxidative tissue injury in clinical samples.^329^ Addressing these gaps will be essential for improving mechanistic understanding and clinical translation.^330,331^

A major theme emerging from this literature is the pronounced context dependence and dual biological roles of both pathways. In cancer, induction of cuproptosis or ferroptosis may eliminate therapy-resistant cells by exploiting their metabolic vulnerabilities. However, ferroptosis may also promote a pro-metastatic inflammatory microenvironment in certain settings. Their immunogenic potential further positions these pathways as promising partners for immunotherapy.

In infectious diseases, ferroptosis may contribute to host defense but can also be exploited by pathogens to facilitate immune evasion. Another important conclusion is the close relationship between these death pathways and cellular metabolism. Sensitivity to cuproptosis and ferroptosis is shaped by metabolic state, lipid composition, and antioxidant capacity. This metabolic dependence helps explain their relevance to metabolic disorders and identifies additional opportunities for therapeutic intervention.

Despite strong preclinical promise, clinical translation remains challenging. Negative outcomes from iron chelation trials in Alzheimer’s disease and Parkinson’s disease illustrate the risks of systemic and nonspecific modulation of essential metals. These findings emphasize the need for more selective and nuanced therapeutic strategies. Future progress will depend on development of advanced delivery systems, including nanoparticles and antibody-drug conjugates, to achieve cell- and tissue-selective effects. Identification of robust biomarkers for patient stratification and response prediction will also be essential for successful implementation within precision medicine. Integration of multi-omics, single-cell technologies, and artificial intelligence-based analytical approaches may further improve patient selection and treatment monitoring.

Three specific research directions emerge from this synthesis. First, dual-metal targeting strategies that simultaneously modulate copper and iron metabolism may exploit metabolic vulnerabilities in therapy-resistant cells. Second, clinical biomarker panels are required to reflect the functional state of cuproptosis and ferroptosis pathways. These panels may include lipoylated protein levels, labile iron pools, lipid peroxidation products, and markers of mitochondrial injury. Such biomarkers would improve patient stratification in future trials. Third, single-cell and spatial multi-omics technologies should be applied to define heterogeneous cuproptosis and ferroptosis responses within the microenvironment. This approach may facilitate more precise combination regimens that integrate metal-dependent cell death inducers with immunotherapies.
